# Putting Theory to the Test: Which Regulatory Mechanisms Can Drive Realistic Growth of a Root?

**DOI:** 10.1371/journal.pcbi.1003910

**Published:** 2014-10-30

**Authors:** Dirk De Vos, Kris Vissenberg, Jan Broeckhove, Gerrit T. S. Beemster

**Affiliations:** 1 Molecular Plant Physiology and Biotechnology, Department of Biology, University of Antwerp, Antwerp, Belgium; 2 Plant Growth and Development, Department of Biology, University of Antwerp, Antwerp, Belgium; 3 Computational Modelling and Programming, Department of Mathematics and Informatics, University of Antwerp, Antwerp, Belgium; Johns Hopkins University, United States of America

## Abstract

In recent years there has been a strong development of computational approaches to mechanistically understand organ growth regulation in plants. In this study, simulation methods were used to explore which regulatory mechanisms can lead to realistic output at the cell and whole organ scale and which other possibilities must be discarded as they result in cellular patterns and kinematic characteristics that are not consistent with experimental observations for the *Arabidopsis thaliana* primary root. To aid in this analysis, a ‘Uniform Longitudinal Strain Rule’ (ULSR) was formulated as a necessary condition for stable, unidirectional, symplastic growth. Our simulations indicate that symplastic structures are robust to differences in longitudinal strain rates along the growth axis only if these differences are small and short-lived. Whereas simple cell-autonomous regulatory rules based on counters and timers can produce stable growth, it was found that steady developmental zones and smooth transitions in cell lengths are not feasible. By introducing spatial cues into growth regulation, those inadequacies could be avoided and experimental data could be faithfully reproduced. Nevertheless, a root growth model based on previous polar auxin-transport mechanisms violates the proposed ULSR due to the presence of lateral gradients. Models with layer-specific regulation or layer-driven growth offer potential solutions. Alternatively, a model representing the known cross-talk between auxin, as the cell proliferation promoting factor, and cytokinin, as the cell differentiation promoting factor, predicts the effect of hormone-perturbations on meristem size. By down-regulating PIN-mediated transport through the transcription factor SHY2, cytokinin effectively flattens the lateral auxin gradient, at the basal boundary of the division zone, (thereby imposing the ULSR) to signal the exit of proliferation and start of elongation. This model exploration underlines the value of generating virtual root growth kinematics to dissect and understand the mechanisms controlling this biological system.

## Author Summary

The growth of a plant root is driven by cell division and cell expansion occurring in spatially distinct developmental zones. Although these zones are in principle stable, depending on the conditions, their size and properties can be modulated. This has been meticulously described by kinematic studies, which have led to the proposal of mechanisms underpinning those observations. At the same time, much knowledge of the identities and interactions of molecules involved in these mechanisms has accumulated, in particular from the model species *Arabidopsis thaliana*. Here we attempt to resolve the longstanding question whether observed growth patterns can be explained by autonomous decision-making at the level of individual cells or if the aid of some external signal is required. We then ask, building on the accumulated molecular information, which minimal models can provide for stable growth while keeping sufficient flexibility to regulate growth. Therefore, we constructed computational models for different growth mechanisms operating in a virtual two-dimensional *Arabidopsis* root and compared their behaviour with biological experiments. The simulations provide strong indications that spatial signals are required for realistic and flexible root growth, likely orchestrated by the plant hormones auxin and cytokinin.

## Introduction

Regulation of plant growth and development has been the subject of intensive research for over a century and this will likely continue, given the increasing need for crop production to sustain a growing world population [1]. By virtue of new experimental approaches *Arabidopsis thaliana* has surpassed classical crops like wheat, tobacco and maize as the main model system to study the underlying molecular processes, thereby considerably advancing the field [2]. The principal processes that determine the growth of plant organs, including the primary root, are cell division and cell growth. Detailed analyses of these processes at high spatial and temporal resolution can be obtained by means of kinematic analyses providing a link with whole organ growth measurements [3], [4]. Under consistent growth conditions, typically around six days after germination the *Arabidopsis* seedling root has established a steady root growth rate. At the distal end of the root a stable zone of cell proliferation (division or proliferation zone: DZ or PZ, respectively) precedes a delineated zone of accelerated growth (elongation zone, EZ). Cells in the elongation zone grow anisotropically reaching 20-fold length increases in as little as six hours, by massive vacuolar expansion, before reaching their final length (in the mature zone, MZ), where root hairs in specific epidermal cell files (trichoblasts) indicate a final differentiation [5], [6]. This relatively simple outline of the growing root apex originates from a more complex underlying organisation. Arranged concentrically around the longitudinal axis, the root apex contains well-defined cell layers originating from stem cell-like initials organized in a tiered arrangement around a zone with low mitotic activity (the quiescent centre, QC; [7]). These initials give rise to 6 distinct cell layers in the proximal direction: stele, pericycle, endodermis and cortex (from initials proximal to the QC), epidermis and lateral root cap cells (from initials lateral to the QC), and one in the distal direction: columella cells (from initials distal to the QC). How the underlying tissue structure is established and maintained remains the subject of extensive investigation [8].

As ample evidence demonstrates, plant hormones play a central role in growth and development and they enable plants to respond to changing circumstances [9]. The wealth of molecular knowledge has inspired various attempts to capture aspects of those complex phenomena in mathematical models (reviewed in [10], [11]). Some modelling studies have focused on root growth. The landmark study of Grieneisen *et al.* [12] used experimental findings on PIN auxin-exporter distributions to reproduce realistic auxin patterns in the root apex that govern cell division and growth. Laskowski and coworkers [13] extended this model to account for lateral root initiation based on auxin maxima induced by root bending. Swarup *et al.* [14] and Band *et al.* [15] used modelling approaches to link asymmetric auxin redistribution at the root tip to the gravitropic response. Mironova *et al.* [16] presented the so called ‘reflected flow’ mechanism as an alternative to the ‘reverse fountain’ mechanism [12] to explain auxin self-organisation, especially during the very early stages of root development. Cruz-Ramírez *et al.* [17] proposed models that integrate radial patterns of the transcription factors SHR and SCR with longitudinal information from an auxin gradient to control asymmetric cell divisions required for differentiation within the *Arabidopsis* root. With a recent spatial model of the root tip Santuari *et al.* [18] implicate differential endocytosis in explaining how the auxin gradient is interpreted throughout the meristem. Band *et al.* [19] have modelled root growth as a single file of cells with growth-driven dilution of gibberellins providing a mechanism for growth arrest toward the end of the elongation zone.

Although some of those models comprise cell growth and division, these processes are typically implemented in an arbitrary and artificial way since their relationship with hormone action has not been sufficiently elucidated. Moreover, plant cells are connected mechanically through their cell walls, which by their stiffness counteract the hydrostatic turgor pressure driving cell growth. The collective framework of growing cell walls (apoplast), imposes major constraints on organ growth, preventing neighbouring cells to become separated (precluding for instance ‘cell sliding’). This type of growth, designated ‘symplastic’, [20], has only recently been represented in a vertex-based computational model of root growth [21]. By creating mechanical stresses, in principle the apoplast operates as an integrative function in coordinating organ growth [22]–[24]. In this study we explore the prowess of previously proposed mechanisms for regulation and coordination of growth and division in a symplastic framework by building and testing computational models within the simulation platform VirtualLeaf [25]. The simulation results are discussed in relation to classical kinematic studies of *Arabidopsis thaliana*.

## Results

### Model construction and evaluation

**Figure 1:**
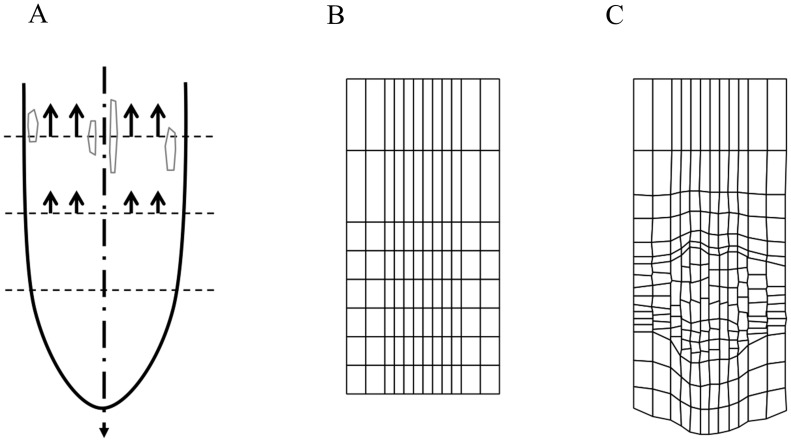
Importance of radially uniform (longitudinal) strain rates. (A) Schematic representation of the ‘ULSR’. Growth of the root apex is simplified as occurring strictly vertically. Arrows indicate that local strain rates are the same at each position along the vertical axis. In our modelling framework one horizontal axis can intersect cells with various average positions, sizes and strain rates thereby inevitably challenging the ULSR. (B–C) Simulation output of *Model 1* (Table S1) with growth defined as a constant increment to a cell's target area. Cells in inner files are narrower and therefore longitudinal strain will be higher. This leads to growth irregularity with faster growth for central files creating an imbalance in the observed strain rates and distortion of the originally horizontal cell walls across the diverse layers. (B) Starting state of cell grid. (C) Cell grid depicted at simulation time 108 h.

Kinematic growth analyses aim to infer properties of cell growth and division from microscopic time series of the growing root, yielding detailed profiles of cell expansion (extension) and cell division (partitioning) along the principal axis of growth [3]. Among others Green [26] developed and advocated this method. Interestingly, Green also derived how proposed elemental growth models would be manifested in a kinematic framework. Based on our current concepts of growth regulation we have extended this analysis that relates changes in cell size along the root axis to relative rates of cell division and expansion. To this end we used the vertex-based plant modelling software VirtualLeaf [25] to directly simulate a selection of distinct growth models (see Methods). These models evolve a two-dimensional cellular grid that represents an axial bisection of the growing root apex (Figure 1A and 1B). Cells are defined as polygons and cell wall segments correspond to the edges acting as linear springs. Cells and cell walls are endowed with biochemical properties represented by reaction and transport equations and logical rules. Specifying regulatory mechanisms ( = input) amounts to specifying rules that minimally determine (i) cellular growth rates (via changing target areas), (ii) cell division rates and orientation (here strictly horizontal), (iii) the transition at the border of the QC and the proximal meristem, (iv) the transition between division and elongation zone (DZ and EZ, respectively), and (v) the transition to mature (differentiated) cells. By modifying those rules and analysing the resulting virtual phenotypes in terms of microscopic and kinematic characteristics we then delineate the requirements for cell growth and division needed to produce a realistic and stably growing root. We have opted here to look at proposed elementary mechanisms and gradually increase the degree of realism compared to real data.

Cell division in plants is spatially confined to meristematic zones, which has inspired discussions on whether its regulation takes place at the supra-cellular (‘organismal’) level instead of the level of individual cells (‘cellular’) [27]. Although as often the truth probably lies somewhere in between [28]–[30], we here classify regulatory mechanisms as cell-autonomous or non-cell-autonomous [31]. In the first case cells behave as pre-programmed automata and there is no role for spatial signalling. In the second case regulation relies on spatial cues that are a function of the organisation of the symplast and that feed back into the cellular decision centres. Here we will define and evaluate the outcome of simulations of models corresponding to those two classes of mechanisms.

### Regulatory mechanisms must conform to the ‘Uniform Longitudinal Strain Rule’

A consequence of the symplastic growth of the root is that at a given distance from the tip all cells have the same relative expansion rate [32]. As stated by Ivanov [33], any observed difference in cell lengths between tissues must therefore reflect differences in cell proliferation (see also [26]). Inversely, any form of growth regulation that results in different elongation rates for cells at the same distance from the tip would disrupt symplastic growth (Figure 1A). For instance, suppose all cells at the same (vertical) position in a downward growing root have the same absolute (areal) expansion rate, irrespective of their size (*Model 1*, Tables 1 and S1). With inner cell files narrower than outer cell files (similar to the real root) this fixed size increment results in consistently larger relative elongation rates for the inner tissue layers leading to tissue distortion and unbalanced distribution of mechanical stresses (Figure 1B and C). Note that the same situation would occur when adjacent files contain cells of similar width, but different lengths growing at the same absolute rates. Hence, non-uniform relative strain rates at some position along the principal growth axis eventually lead to malformations.

**Table 1:** Compact model overview.

Model	Fig.	Exit DZ -Start EZ	Exit EZ – Start MZ	Division rule	Growth rate
1	1C	Counter	Timer	Timer	Constant
2	3A–D	Timer	Timer	Timer	Linear
3	S1A	Counter	Timer	Timer	Linear
4	4A	Timer	Timer	Timer+noise	Linear
5	4B	Counter	Timer	Timer (variant)	Linear
6	4C	Counter	Timer	Sizer (uniform)	Linear
7	S3B	Counter	Timer	Sizer	Linear
8	5A–D	Ruler	Ruler	Sizer	Linear
9*	6A–D	Ruler	Ruler	Sizer	Linear
10	7A,B	Auxin threshold	Auxin threshold	Sizer	Linear
11	8	Layer-specific auxin threshold	Layer-specific auxin threshold	Sizer	Linear
12	9	SHY2 threshold	GA threshold	Sizer	Linear

*Model 9 differs from Model 8 in that auxin was added as a model variable to study its dynamics in a realistic, expanding cellular grid.

Overview of the models used in this study. Various categories w.r.t. developmental decisions are presented. Column (3) specifies the transition between division and elongation zone (DZ and EZ, respectively); column (4) specifies the transition to mature (differentiated) cells (mature zone or MZ) based on timing since the release from the QC or a spatial signal at a fixed distance from the root apex; column (5) specifies whether division rate is determined via a timer or sizer mechanism; and column (6) how cellular growth rates are defined. Developmental events can be determined to happen after a fixed duration (‘Timer’), a fixed number of divisions (‘Counter’), a fixed cell size (‘Sizer’), and a fixed distance from the root apex (‘Ruler’). For Models 10–12 more complicated regulatory mechanisms are specified. In Model 4 extra random noise was added to the timer (‘+ noise’). For Model 5 cell division is dependent on a timer mechanism, with a different cell cycle time for inner and outer layers (‘Timer (variant)’). Model 6 uses a uniform size (area) criterion for cell division, irrespectively of cell geometry (‘Sizer (uniform)’). For Model 7 the size criterion is adapted to the difference in the width between inner and outer layers, thereby acting effectively as a length criterion. In the other (non-cell autonomous) cases the cell division sizer differs between layers. It is usually assumed that DZ and EZ have a characteristic elongation rate. More details of the models can be found in Table S1, Text S1 and in the corresponding figures.

Disruption can be manifested in different forms ranging from small local cell or tissue deformations up to changes of whole organ growth. In fact, ‘disruption’ may be an overly negative term as it could be argued that a carefully-coordinated breach of that principle, as for instance in the root gravitropic response, can be beneficial to the plant. Furthermore, the stated necessary condition might be too stringent since small and short-lived random perturbations are likely to yield no significant distortions since they can cancel each other out to some extend as demonstrated in the next section. The apoplast, by its ability to transmit mechanical stress, may effectively function as a buffer to those small and random perturbations. Nevertheless, it seems evident that systematic differences in strain rates will eventually result in geometrical changes to the organ structure. In order to evaluate whether growth mechanisms allow for stable root growth we therefore reformulate the previously mentioned findings of Ivanov [33] and Green [26]:

***If in a uni-directionally growing root at least two points at the same axial position (with respect to the growth direction) have a persistently different longitudinal strain rate (relative elongation rate) then the symplastic structure will be distorted.***

‘Persistent’ should here be interpreted as present during a minimum time interval sufficient to produce an arbitrary distortion based on that local strain rate difference. From here on, we will refer to this formulation as the ‘Uniform Longitudinal Strain Rule’ (in short ULSR) to emphasise its application over a finite time interval. Although root growth of the *Arabidopsis* seedling is in general not steady [5] in some conditions during development this is approximately the case [34]. Here we will investigate also how steady growth can be attained and maintained. Like embryonic roots we will therefore start the models from a minimal set of precursor cells (Figure 1B). Subsequently the models will evolve to a situation where a larger population of cells occupies the meristem, elongation and mature part of the roots. In this process the growth of the root will accelerate initially, but then ideally converges to a state where a constant size of the meristem and elongation zone is obtained and the root growth rate becomes constant. For a uni-directionally growing root the following definition is proposed.

***The growing root is in a steady state if all points at each position along the growth axis have a constant strain rate.***

**Figure 2:**
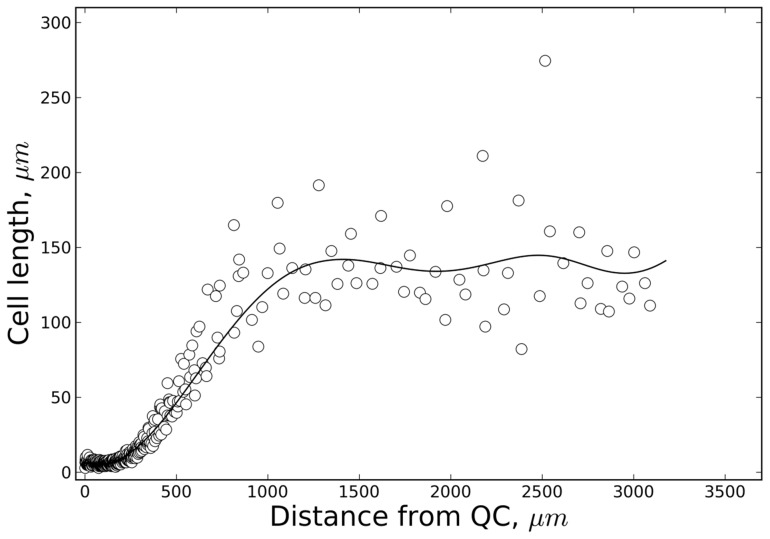
Experimental cell length distribution. Cell length distribution along the principal growth axis (distance from the QC/quiescent centre) determined for a typical 7-day-old *Arabidopsis* seedling root. Experimental set-up and growth conditions were in accordance with Beemster and Baskin [5]. The data points represent epidermal cell lengths. The ‘polyloc method’ was used for curve fitting (full line, *cf.* Methods).

For practical reasons in the next sections our evaluation of stable and realistic growth will be largely based on three simplified criteria:

1. The overall root growth should reach a steady state.
2. The cell length distribution along the root should be similar to what was observed experimentally with:
  + Similar lengths of meristematic and mature cells, connected by a smooth transition [5].
  + An approximately two-fold size range at any position [32].
  Since only averaged data or progress curves have been reported of cell length distributions along the primary root axis of *Arabidopsis*, we have generated a new curve (Figure 2) based on plants cultured in the same growth conditions as in [5].
3. The underlying regulation of cell division and growth should not be in conflict with the ULSR.

### Cell-autonomous regulation does not produce realistic root kinematics

We start off by considering strictly cell-autonomous regulation of cell growth and division as this appears to be the most elementary form of regulation. Cell-autonomous regulation implies here that essential developmental transitions are only governed by endogenous cellular programs independent of any external signals (excluding their direct physical connection in the symplast). Whereas we know this is unlikely the case, some plant growth studies point towards regulation based on cells acting as autonomous, pre-programmed units. Typically, cell behaviour is proposed to be a function of the number of events or the time passed with respect to some reference point (a defined cellular event). These types of regulation are called counters and timers, respectively. A counter keeping track of the number of cell divisions of a cell before exiting the proliferative phase has been repeatedly proposed [34]–[36]. Timers have been used in various growth models for instance on vertebrate segmentation [37] or the cell cycle [12], [38], [39]. It has indeed been found that in wild type roots under diverse treatments, cells spend about the same time traversing elongation zones of very different sizes (even during accelerated growth) suggesting that cells enter the EZ with a pre-programmed duration for expansion activity [5]. In his seminal study Green [26] includes a meristem doubling model in which the meristem consists of a number of cells that undergo one cell cycle after which the proximal half of them enters the non-dividing state.

**Figure 3:**
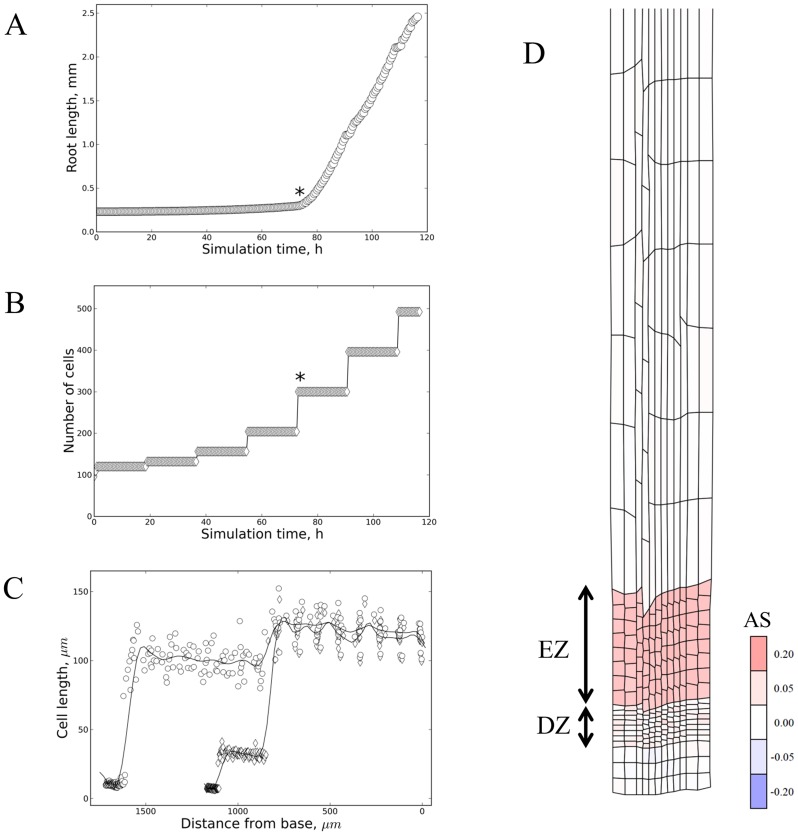
Timers and counters can produce stable directional growth. Example simulations of counter and timer based models for root growth (*Model 2* and *Model 3* in Table S1). Upon release from the QC, cells can divide for a pre-programmed time duration (A–C) or number of times (D), followed by a fixed time duration of accelerated growth. (A) Total root length versus simulation time. It takes approximately 75 hours for the first cells to start accelerating growth (indicated by ‘*’). Subsequent sets of quasi synchronously dividing cells then grow exponentially for a fixed time, yielding a roughly linear area increase. (B) Total cell number versus simulation time. The cell number is built up exponentially till 12 (columns)×2^3^ = 96 ‘clones’ are formed. These then undergo accelerated growth. One cell cycle later the next pool of 96 cells is ready to do the same and cell numbers increase by constant steps. (C) Detailed snapshots (at 99 hours, with diamond shaped markers, and 108 hours, with circular markers) of approximate cell length along the main growth axis. Subpopulations with narrow length distributions can be seen corresponding to dividing, accelerating and mature cells. The accelerating cell population occupies an increasing area until adding to the mature zone. The ‘polyloc method’ was used for curve fitting (*cf.* Methods). (D) Simulation output of *Model 2* at 99 h (Table S1). The imposed growth and division rules have resulted in a highly regular grid with distinct zones of similar cell length (division zone (DZ) and elongation zone (EZ) are indicated). Areal strain rates (‘AS’ as defined in Methods) are mapped on the cellular grid, showing the elongation zone as a distinct region of relatively uniform accelerated growth.

**Figure 4:**
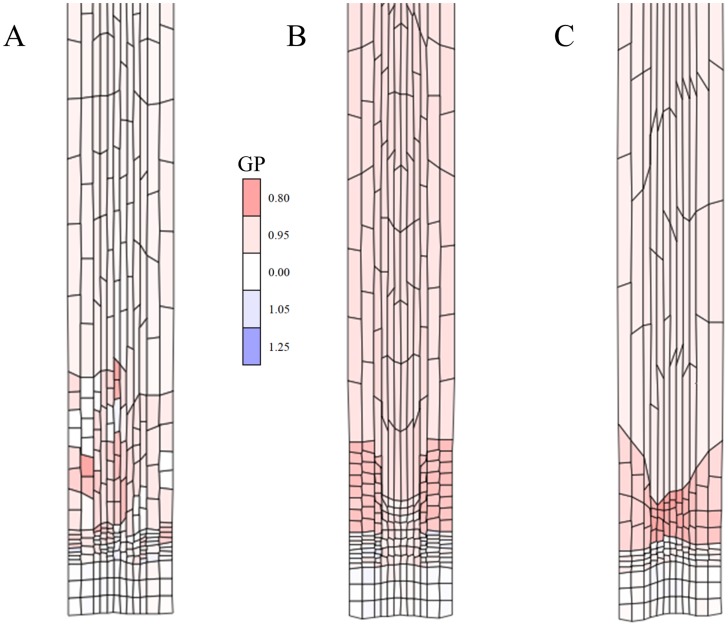
Cell-autonomous regulation: Robustness to perturbations. (A) Simulation output (time: 87 h) from a model as in Figure 3, yet with maximally ±25% noise added to cell cycle time (except first division ±10%, *cf.* Table S1 – *Model 4*; see also Figures S3A and S3C), which demonstrates that the symplast can act as a stabilizing framework that dissipates ‘noisy’ mechanical perturbations through its structure. Colouring is according to growth potential (defined in the Methods section) as a measure for ‘turgor pressure’. (B) Timer-based developmental transitions can amplify tissue distortion. Simulation output of timer mechanism with layer-specific cell cycle time (*Model 5* at simulation time 90 h). Upon release from the ‘QC’ cells can divide 3 times. For the outer 6 cell files CCT = 900 min, the inner 6 CCT = 1080 min. Afterwards, cells undergo accelerated growth until 4440 min after ‘QC’-release. Therefore the inner cells lag behind if their release started more or less synchronous as is the case here (maximally ±10% noise). Synchronicity is an important factor determining stable patterning in itself. Counters are sensitive as they multiply and therefore consistently amplify differences in CCT. Cells in the centre are lagging behind in terms of growth. As simulation time passes the irregularities eventually result in severely distorted patterns. Colouring is according to growth potential (defined in the Methods section) as a measure for ‘turgor pressure’. (C) Simulation of *Model 6* (Table S1) at simulation time 42 h, with a sizer-based cell cycle. One sizer, imposing division at a defined absolute cell size, is used despite differences in width of cells at similar positions along the main growth axis. Outer cell files have wider cells which reach the critical size before those of inner files. Therefore they undergo a much earlier exit from proliferation starting accelerated growth earlier, resulting in cell shape/tissue distortion. Cells in the centre are therefore lagging behind in terms of growth rate. Cell-autonomous regulatory systems appear inherently sensitive to this effect. Colouring is according to growth potential, GP or ‘turgor pressure’ (*cf.* Methods).

To test whether cell-autonomous regulation can produce realistic root apex structures we constructed a set of models in which cells in contact with the columella/QC maintain their capacity to divide. Upon release from the QC, these cells are allowed to divide another two or three times, based on cells keeping track of the number of divisions or the time passed since release from the columella/QC (*Models 2–7*, Tables 1 and S1, Figures 3 and 4). The loss of proliferative capacity is then followed by a fixed time interval of accelerated growth. In *Model 2* (Table 1 and S1) the exit from the proliferative stage is determined by a timer, with cell division synchronous among cells at the same longitudinal position. Starting from a simple grid, after a transient phase, a roughly linear increase in length is achieved in accordance with the first of the proposed criteria (Figure 3A). The synchronized cell division results in regular stepwise increases in cell numbers (Figure 3B). All cells in one file derive from one cell (clones) occurring in groups (of increasing multiples of 2) with the same developmental state (*i.e.* number of divisions and time since QC release). Consequently, discrete sets of developmentally synchronous cells with similar size and growth rate arise (see Figures 3C and 3D) leading to sharp developmental transitions. The use of a counter instead of a timer to decide when cells exit proliferation as in *Model 3* (Table 1 and S1), produces essentially the same outcome (Figure S1). Similarly to what Green described with his meristem doubling model, the size of the proliferation zone periodically varies (Figures 3C and S2), growing exponentially and losing half of its size as a complete group of clonal cells exits proliferation and enters accelerated growth. Importantly, *in vivo* such patterns of synchronised behaviour have not been observed. Contrary to the third (ULSR), the second criterion is breached since cell length distributions along the zones are not smooth (contrary to [5]). Indeed, some aspects of realistic root growth can be reproduced, yet, others not based on this type of model.

Simulating perfectly synchronized cell divisions produces highly regular cellular grids dissimilar to root micrographs (compare Figure S1 with anatomical sections in for instance [7]). After adding random uniform noise (within +/−25% of reference) to each individual cell cycle time a more naturally looking outcome results (*Model 4*, Figure 4A), with a smoother cell number increase in time (Figure S3A). Importantly, in a spatial context the cell length distributions remain discontinuous, yet smeared out more (Figure S3C). Logically with the partial loss of synchronicity, cells at the same position along the growth axis will not necessarily grow at the same relative rate since their number of divisions or the time since release from the QC varies. Hence, the proposed ULSR is violated *sensu stricto*. We have found, however, through our simulations that, to some extent, the symplast acts as a stabilising framework in that local, random mechanical perturbations are dissipated through its structure (Figure 4A, with colouring as a function of the ratio actual area/target area, with red colours representing an outgoing or cell-expanding potential: for details see Methods). However, if even more cell cycle time noise is added or if perturbations are systematic, then growth defects eventually arise. For instance in *Model 5* and *Model 6* (Tables 1 and S1) a systematically shorter cell cycle time of the outer cell layers is propagated over multiple generations. Through timer or counter based rules the cells in the outer layers undergo the fast acceleration phase much earlier, leading to tissue structure defects (Figure 4B and 4C). Cell-autonomous regulation is prone to this since a local defect is easily clonally amplified, especially if occurring at an early stage in development, without external signals to constrain the effect within spatial bounds.

Likely, robust regulation also includes well-coordinated cell division and cell growth, leading to a stable cell size distribution over time. Contrary to a simple pre-programmed cell cycle timer, which does not respond to fluctuations in growth rate, a size-based cell division mechanism (‘sizer’) might provide this extra stability. Size-based control mechanisms are well-established in (plant) cell cycle modelling (*e.g.* [40], [41]. If cell division is implemented to happen at an absolute cell size (or length rather as in *Model 7*) cell sizes (or lengths) are kept in strict bounds in the DZ, yet synchronicity of cell division is reduced (Figure S3B).

We conclude that despite a quasi-steady growth, in the absence of spatial cues stable developmental zones and smooth gradients in cell lengths are not feasible based on the investigated types of strict cell-autonomous regulation. It makes sense that to have cells of the same generation behave according to a smooth positional gradient requires a spatial signal, even in the presence of noise.

### Non-cell-autonomous regulation provides a solid basis for realistic root kinematics

**Figure 5:**
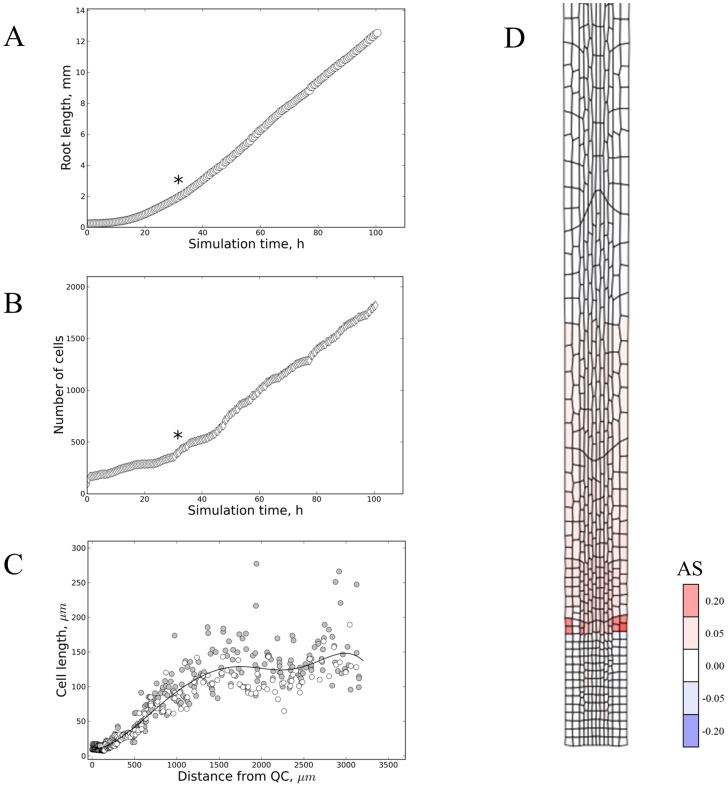
Smooth developmental transitions through spatial signalling. Cells are instructed by spatial signals at a fixed distance from the growing root apex (*cf.* Table S1 – *Model 8*). They do not behave as clonal subpopulations and smooth developmental processes are a natural result. (A) Plot of root length versus simulation time shows a smooth transition to a steady linear organ growth (indicated by ‘*’). This is similar to experimental studies (*cf.* Figure 1 in [34]). (B) Plot of total cell number versus simulation time shows a roughly similar trend as in (A) (‘*’ indicating approximately steady increase). (C) Cell length along the principal growth axis (at simulation time 50 h) demonstrates that the exit of division and start of accelerated growth at a fixed position from the apex can lead to a smooth cell length profile as seen in experimental studies (compare Figure 2). Grey circles represent data points across all cell layers, whereas empty circles are data from the 2 outer (here called epidermal) cell layers only. The ‘epidermal’ data points lie roughly within the expected twofold range at each position along the longitudinal axis. The ‘polyloc’ method was used for curve fitting (*cf.* Methods). (D) Simulation output with areal strain rates (‘AS’ as defined in Methods) mapped on the cellular grid, showing the elongation zone with accelerated growth. This represents a snapshot at 45 h from a model similar to *Model 8* (except a relative growth rate of 0.2 per simulation step of 30 min, occurring between 240–750 µm from the root apex and division at a size of 360 and 180 µm^2^ for outer and inner cell layers, respectively).

*Model 8* (Tables 1 and S1) was constructed to test whether including spatial signals (non-cell-autonomous regulation) can improve resemblance with experimental data. Counter and timer-based rules for exit of proliferation, start of accelerated growth and maturation were replaced by a fully independent spatial signal that marks these transitions at fixed positions from the QC (like a ‘ruler’). Simulations with this concept model lead to growth, which to a good approximation can be described as steady and linear (Figure 5; Video S1). The spatial boundaries directly limit the total mass production occurring within those predefined zones. Importantly, the simulated data of (epidermal) cell lengths along the root apex are similar to what we obtained experimentally (Figure 2), and the fitted cell length distributions are similar to what was reported in previous kinematics studies as well [5]. The strain rates are either low (in the DZ) or high (in the EZ) changing rather abruptly going from DZ to EZ for a single simulated root (Figures S4A). Using averaged strain rates from multiple simulations (with different random seeds for mechanical equilibration) shows that the strain rate curves become more smooth and bell-shaped (although still skewed) as was observed in various studies [42], [5], [43] (Figure S4B). Accordingly, Beemster and Baskin [5] described sharp changes in the slope of the velocity profiles for individual root samples versus averaged data (compare their Figure 2A (including inset) to Figures S4C and S4D). This rapid change was also found by van der Weele *et al.* [44]. Furthermore, since no conflicts with the ULSR appear to exist, non-cell-autonomous regulation can effectively fulfil all proposed criteria for realistic root growth.

**Figure 6:**
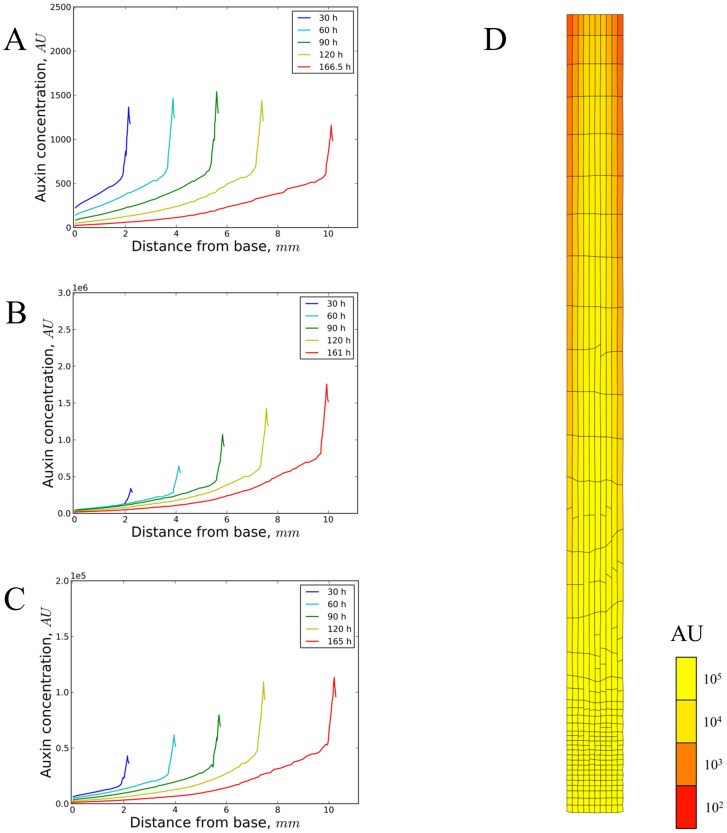
Auxin dynamics on a dynamic cellular grid. Output of model simulations (Table S1 – *Model 9*) with cells growing exponentially (in accordance with [32]) at the same low specific growth rate (except the ‘cap’ cells) up to a fixed distance of 240 µm from the tip and then undergoing 10-fold growth acceleration (up to a specific distance of 750 µm). Cell division was based on a sizer mechanism with noise added to have a more irregular, natural, tissue shape (up to a fixed distance of 240 µm from the tip). To not bias the analysis, growth and division did not depend on auxin. Four simulations based on differences in kinetic parameter values as well as some subtle differences in the differential equations were set up to represent different types of auxin sources in the model (see also Text S1, Figure S5). (A–C) Auxin profiles over time in the growing root. (A) A strictly external source of auxin (import from upper vascular and border cell walls, export via epidermal cell walls). Kinetic parameters (*cf.* Text S1): 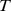 = 1 µm; 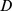 = 6000 µm^2^.min^−1^; 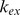 = 1200 µm.min^−1^ 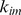 = 600 µm.min^−1^; 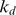 = 0.0003 min^−1^; 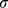 = 0 (µm^2^ min)^−1^; 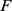 = 2.10^7^ min^−1^. The sharp peak at the apex shifts and slowly dilutes out by steady growth. (B) A strictly internal auxin source (production rates proportional to cell areas). Kinetic parameters that are different from (A): 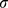 = 100 (µm^2^ min)^−1^; 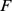 = 0 min^−1^. The shape of the curve is as in (A) without peak dilution. (C) A strictly internal auxin source (production rates constant on a per cell basis). Kinetic parameters that are different from (A): 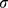 = 10^4^ min^−1^; 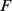 = 0 min^−1^. A similarly shaped curve is shown as (A) and (B), but with peak convergence as time progresses. (D) Steady auxin concentration pattern (yellow colouring according to arbitrary concentration units ‘AU’) on a dynamic cellular grid (simulation time 30 h, growth according to the rules in Table S1 – *Model 9*), with both external and local (area-dependent) auxin sources (and sinks). Kinetic parameters: 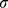 = 100 (µm^2^ min)^−1^; 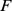 = 2.10^7^ min^−1^. Figures S6, S7, S8, S9 illustrate the dependence of the shape of the auxin gradient on the parameters used here (in a non-growing root). More information on the kinetic equations can be found in Text S1.

From a biochemical point of view the most likely spatial signal to act as a ‘ruler’ would be a phytohormone or combination of phytohormones undergoing long-distance transport through the root tissue resulting in a morphogenetic gradient. Auxin is arguably the most prominent root morphogen. It has been demonstrated that a stable auxin gradient can be formed which could act as the primary signal determining growth and cell division [12]. In the simulations reported with that model the growing root tip is regularly truncated providing an auxin source at a constant distance from the root tip. Via *Model 9*, which is a vertex-based variant of the latter model (Table 1 and S1) we investigated whether auxin patterns effectively reach a steady state with a source at a fixed position that becomes displaced further and further from the root tip due to growth. We specifically explored how spatially regulated growth and division (as in Figure 5) affect the auxin gradient (not conversely). It followed that an important factor is the definition of the auxin source: as a (constant) net import from the upper plant parts (and) or as some form of local production. In the first variant of *Model 9* (in the presence of first order auxin degradation) only the total auxin level of the root slowly converges to a steady state (Figure S5A), whereas the concentration is steadily diluted (after an initial increase) by the growing root (Figure S5B and S5C). If we define developmental transitions with a stable spatial signal, therefore this type of auxin source in principle does not support steady root growth. Rather, it might be suitable to produce temporary responses. A different behaviour emerges with variants of *Model 9* that instead use local (root-based) auxin production: either with cellular production proportional to size or with a constant production rate per cell. In both cases the total auxin level (Figure S5D and S5G) increases proportionally to the area increase (Figure S5E and S5H), and the auxin concentration over the total root slowly (especially for the area-based production mode) converges to a steady state (Figure S5F and S5I). Production modes can be combined as well, yet this does not change the picture with typically one mode being (or becoming) dominant (results not shown). In accordance with previous work, the two-dimensional shape of the auxin gradient depends on whether a-polar (diffusive or AUX/LAX importer-mediated) or polarized (PIN-mediated) transport is dominant, with in the first case a gradual auxin concentration increase towards the lower (distal) root combined with a smooth auxin peak near the ‘QC’ cells (Figure S6). If PIN-transport is dominant a much more pronounced local auxin maximum occurs at the central cell lines just proximal to the ‘QC’ cells. The dominance of diffusive versus a-polar transport is determined in this model by the relative values of the diffusion and PIN transport kinetic constants rather than their absolute values (see for instance the similarity of Figures S6A and S6E, and Figures S6B and S6F). With the model variant based on constant auxin import that sharp peak shifts in time, connected to the growing root apex. However, it eventually disappears by growth dilution (Figure 6A). Local production modes again show the shifting of the sharp peak, however, in those cases the sharp peak slowly but gradually becomes stable (Figure 6B and 6C). We conclude that typical auxin-based models can produce stable gradients if auxin production scales with the growing root. Importantly, unless auxin transport is completely a-polar, there is always an additional horizontal (transversal) concentration gradient besides the longitudinal gradient (Figures 6D, S6, S7, S8, S9). This corresponds to what can be seen in various reporter studies [18], [45]. How this relates to growth regulation is investigated in the next section.

**Figure 7:**
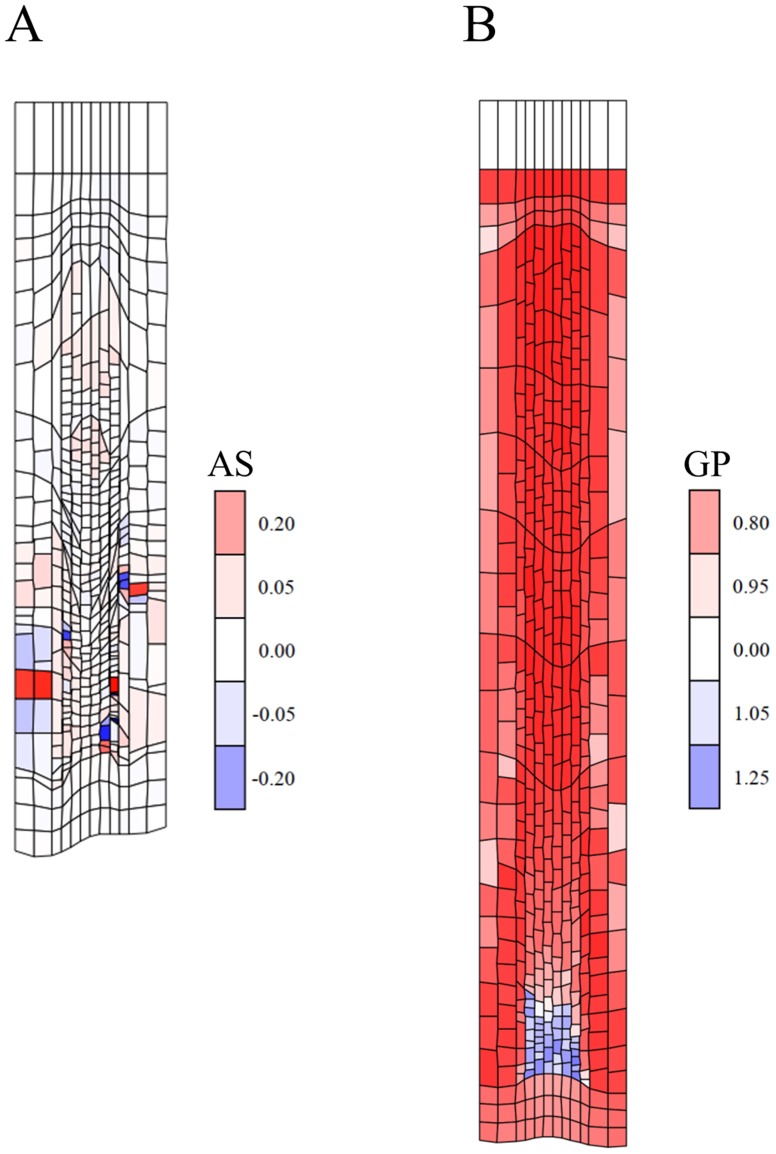
Morphogen-regulated growth and division can violate the ULSR. (A) Snapshot of a simulation (simulation time 84 h) from an auxin-based developmental model (*Model 10*, Table S1) in which cell division and (slow) growth are only possible above a fixed threshold of auxin concentration. Below this threshold (13.5 AU) and above a second lower threshold (8.8 AU) cells undergo accelerated growth. From an early stage of growth on strain rates are unbalanced leading to tissue distortion. The malformations accumulate and cell divisions are predominantly taking place in the central layers as determined by the auxin gradient. Colouring according to areal strain rates (‘AS’, *cf.* Methods) (B) Snapshot of a simulation with *Model 10*, but with a more dominant diffusion regime (parameters as in Figure 6D, with as threshold for accelerated growth <60000 AU and for growth termination <40000 AU) leading to a less pronounced lateral gradient (at 90 h). This produces less severe tissue distortion, but still severely inhibits growth and leads to unrealistic cell size distributions. Colouring is according to growth potential (‘GP’, as defined in the Methods section) as a measure for ‘turgor pressure’, showing a central region at the apex which opposes growth of the outer cell layers (indicated by blue versus red colours).

Having obtained a steady and stable spatial auxin gradient, the question remains how it is interpreted by the cells as they move through it. In *Model 10* (Tables 1 and S1) cell growth and division are directly determined by the local auxin concentration, *e.g.* according to specific step functions (division above a minimum size and above an auxin threshold, slow growth above the same auxin threshold, fast growth above a lower threshold). In this case no steady state was obtained with severely distorted root growth (Figure 7A). Because of the radial gradient, cells at the same position along the growth axis are instructed to consistently produce different strain rates thereby violating the ULSR. With auxin concentration determining cell division, cell size distributions are not arranged in parallel along the longitudinal axis but follow the peaks and valleys in the auxin landscape (to some extent this has been observed, (*e.g.* [46]). When tuning the auxin transport parameters to a more dominant diffusion regime the transversal gradient remains present still precluding stable growth, although the distortions of the cellular grid are less pronounced. Some tissue locations (for instance the vascular layers near the QC) have a much lower tendency to grow (blue colours) compared to others (red coloured outer layers near the QC) and inhibit and distort growth (Figure 7B). Alternative scenarios based on spatial or temporal derivatives of the auxin concentration pattern (as proposed for instance for *Drosophila* organ development [47]) do not provide more regularly spaced developmental cues (results not shown). We conclude that in itself a strictly auxin-based growth model is unlikely to produce stable and realistic root growth. Whereas a simple diffusion-based auxin transport mechanism (*e.g.* with a source near the ‘QC’) could resolve this issue, this spatial complexity resulting from polar auxin transport is important for other purposes, like auxin's role in regulating formative divisions around the ‘QC’ [17]. In the following we investigate two opposing theories proposed to circumvent this differential growth problem in response to a lateral (radial) auxin gradient.

**Figure 8:**
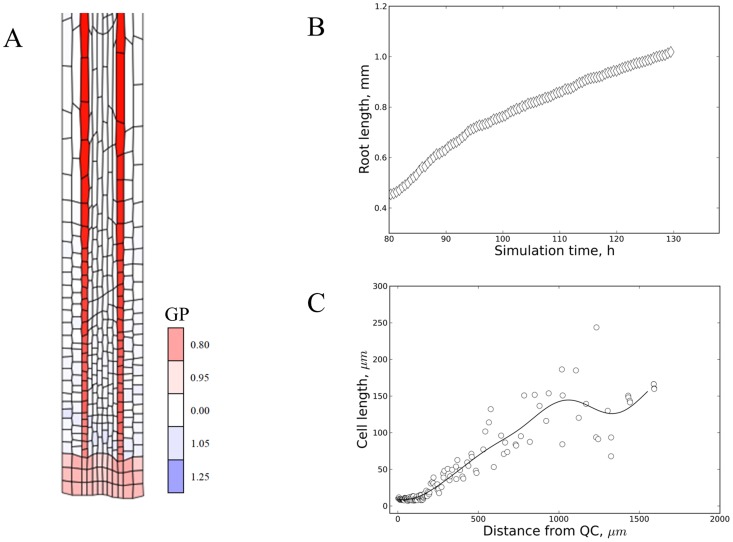
Layer-driven growth can alleviate problems with the ULSR. (A) Layer-driven auxin-dependent growth according to *Model 11* (Table S1). Simulation time 109.5 h of model for which auxin concentration is ‘interpreted’ by the two layers of border cells (in analogy with endodermis-specific growth regulation by GA [78], a different tissue layer, for instance the epidermis, could be equally effective: result not shown) and translated into an increase in the target area of those cells (*cf.* Methods). The other cell layers are programmed to follow passively by re-setting their target areas to their actual areas after every simulation step in accordance with a small resisting force w.r.t. the layer that is controlling growth. Colouring is according to growth potential (‘GP’, as defined in the Methods section) as a measure for ‘turgor pressure’, showing border cells drive growth of neighbouring cells to the extent that their target areas are smaller than their actual areas (slight blue colour). (B) Plot of root length versus simulation time shows steady linear organ growth from 94 h on after a long preparatory phase to construct a realistic starting grid with a stable auxin gradient (code details in Dataset 1). (C) Plot depicting the cell length along the principal growth axis at step 103.5 h of the simulation with a model equivalent to *Model 8* but with the growth driven by the 3th and 10th layer as in *Model 11*. Note that cell lengths vary smoothly from DZ to EZ similar to Figure 2 and 5C.

It has been recently highlighted that hormones seem to regulate root growth in a layer (/tissue)-specific way [9]. Whereas short-range (paracrine) signals have been put forward as a hypothetical solution to transmit developmental signals transversally through the layers [9] we have tested whether it is sufficient for cell layers to ‘communicate’ through their mechanical connections. *Model 11* (Tables 1 and S1) represents this concept of ‘layer (/tissue)-driven growth’ by selecting one arbitrary cell file (represented by 2 cell columns) in which the cells undergo turgor-driven growth. The other cell files are forced to passively follow (Figure 8, see legend and Dataset S1 for implementation). Simulations show that one cell file can effectively drive steady growth of the whole organ, yielding smooth cell length distributions (Figures 8B and 8C). Strictly, a conflict exists with the ULSR since one cell file is consistently growing faster. Nevertheless, if the cells in the other layers can respond rapidly (adjusting their target areas or ‘turgor pressures’) then the difference in strain rate apparently remains sufficiently low to cause much overall tissue distortion. This rapid response requires transversal communication between the layers. Biologically, signal transduction could potentially be mediated by the mechanical connections between the layers or otherwise a secondary chemical signal would have to be transmitted. Irrespectively, we conclude a layer-driven mechanism can solve the problem of layer-specific differences in the instructive hormone (here auxin) gradient.

**Figure 9:**
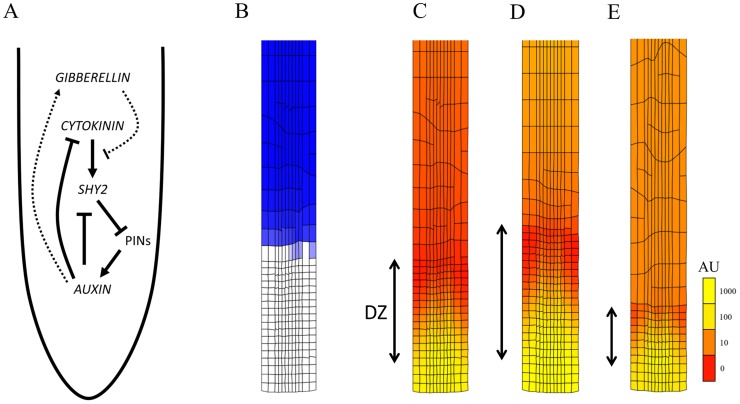
Cytokinin-auxin cross-talk in root development. Simulation output of *Model 12* (Table S1). (A) Schematic view of regulatory interactions between model variables (in *italics*) and PIN exporters. Dotted lines illustrate potential cross-talk with gibberellin (GA) signalling (auxin stimulating GA and GA inhibiting cytokinin signalling) not included in the model. GA is represented in the model as an independent signal that undergoes growth-dilution, thereby determining the exit from elongation [19]. (B) Simulation output at 30 h with blue colouring relative to the SHY2 concentration. A domain with strong SHY2 expression is present. (C–E) Colouring of the cell grid is according to the auxin concentration in arbitrary units (‘AU’). Notice a transition from a (basal) linear gradient to a (apical) 2D gradient dominated by polar transport. This is caused by the PIN inhibition at the SHY2 expression domain. The extent of the division zone (DZ) is indicated. (D) Simulation of this model with a 4-fold stronger auxin source shows that the DZ is expanded. (E) Simulation of this model with a 4-fold stronger cytokinin source shows that the DZ has shrunk considerably. This corresponds to observations from Beemster and Baskin [34] on treatment of *Arabidopsis* root with auxin and cytokinin analogues.

A different solution to reconcile the central role of auxin in root growth with the ULSR may consist of introducing regulatory cross-talk into the models. In other words to complement or modulate the auxin signalling other spatially non-uniform signals, like transcription factors [17], [48] or other hormones [49]–[52] are implicated. Based on proposed regulatory interactions [53], [54], we have implemented a model (*Model 12*, Tables 1 and S1, Text S1) centred around the cross-talk between auxin as the cell proliferation promoting factor and cytokinin as the cell differentiation promoting factor (Figure 9A). Auxin transport was described as both diffusive and PIN-mediated, with local production and an external source as in the previously discussed models. Cytokinin transport was described as strictly diffusion-based, also with local and external sources, the local production rate being repressed by auxin. This led to a shallow gradient, the concentration decreasing towards the tip (as in [55]). In this simplified model cytokinin up-regulates the transcription factor SHY2 which is typically strongly expressed at the so called transition zone between DZ and EZ [54]. Increasing SHY2 leads to repression of PIN transporter levels and a resulting auxin signal drop that reduces the meristem size. Accordingly a local auxin response minimum has been observed before [18]. Auxin on the other hand has been proposed to counteract this effect by mediating SHY2 degradation, in part through gibberellin (GA) repressing ARR1 via the DELLA family protein RGA. In our simulation model we did not include any ARRs as variables. In accordance with others (see for instance [56]) auxin was assumed to directly inhibit cellular cytokinin production. GA was only included as a variable that determines where the EZ ends due to simple growth dilution (in accordance with [19]). The exit from proliferation and start of accelerated growth was determined by crossing a SHY2 concentration threshold (*cf.* Text S1). Interestingly, because of its inhibition of PIN expression, the steep increase in the SHY2 concentration coincides with a dip in the auxin concentration as well as the transition from a strongly polarized to a predominantly a-polar transport regime (Figures 9B and S10A) leading in general to much more linear spatial gradients in accordance with the ULSR (Figures 9C–E and S11). Since the first two proposed criteria for realistic growth were fulfilled too (Figures S10B,C), this mechanism in principle allows for the required regular and stable zonation of the growing root apex, and can therefore potentially reconcile multiple roles for auxin in patterning along different directions. Furthermore, with *Model 12* we could qualitatively predict how adding external auxin and cytokinin affects the meristem size (increasing and decreasing in size, respectively) as demonstrated experimentally by Beemster and Baskin ([34]; Figure 9C–E). We conclude that this computational model most effectively captures the basic growth characteristics of the *Arabidopsis* root and represents an ideal starting point to develop more advanced computational kinematic models which can predict root growth under more diverse conditions and perturbations.

## Discussion

We have constructed and simulated different models that represent steady symplastic growth of the *Arabidopsis* root tip. We compared diverse regulatory mechanisms and found out which of them can adequately reproduce crucial properties of primary root growth, according to three well-defined criteria (steady-state, realistic cell length distribution, and ULSR) that allow rigorous comparison with experimental observations of *in vivo* growing roots of *Arabidopsis thaliana*. A necessary condition for stable unidirectional symplastic growth [33], [26] was re-interpreted and re-formulated as a strain (rate) rule (‘ULSR’) for those mechanisms to conform to. It is mainly this third criterion that warrants the use of our sophisticated vertex-based simulations rather than the more simple approach of modelling a single cell file such as in [19], which could potentially produce kinematic output such as steady state length growth and cell length profiles. As soon as differences in strain rates or complex transport phenomena occur, the use of a two-dimensional tissue representation becomes necessary. Our simulations do suggest that symplastic structures are resilient to differences in strain rates as long as they are small and short-lived. How far or fast perturbations are precisely transmitted through plant tissue and to which extend this affects organ growth are intriguing questions that go beyond the scope of this study. A more accurate representation of cell wall mechanical properties (such as [21]) and of tissue and organ structure (possibly in 3D) would then be desirable and would further enhance kinematic and microscopic resemblance with real root tissues.

Considering the complexity of molecular interactions implicated in growth regulation of the *Arabidopsis* root apex and considering the presence of many gaps in our understanding, we have opted rather to define rules for cell growth and division inspired by previous studies and enriched them with molecular interactions whenever useful. Whether regulation takes place at the supra-cellular (‘organismal’) level versus at the level of individual cells (‘cellular’) forms the subject of a long standing discussion [27]. This distinction is probably overly polarized as indicated by experimental observations [29]–[31]. In fact, signals that vary on the cellular, tissue- and organ-scale are known to affect pattern formation considerably. We have kept this conceptual distinction nevertheless and classified the proposed regulatory mechanisms as cell-autonomous (based on local pre-programmed rules) or non-cell-autonomous (affected by external, spatial signals).

Cell-autonomous mechanisms like timers, counters and sizers are readily implemented with the logical expressions of a computer program. In a biological context such mechanisms typically require more complex designs [57]–[59]. Various sizer and timer based cell cycle models have been reported, making it reasonable representations of cell behaviour [38]–[40]. Given the conserved nature of the eukaryotic cell cycle machinery these types of mechanisms are likely operating in the plant cell cycle [41], [60]. Timers and counters operating at different spatial and/or temporal scales are even more obscure. For instance, a developmental counter that determines the exit of proliferation would have to keep track of the number of cell divisions a cell has undergone. The epigenetic state of a plant cell can reflect this history [61]. In the context of plant development, telomere shortening could play such a role [62]. After cells exit the DZ, DNA duplication cycles are expected to continue through the process of endoreduplication. The number of DNA copies could therefore serve as a direct developmental marker to the plant cell.

We have shown that stable growth is possible based strictly on counter and timer mechanisms. However, the absence of spatial cues precludes realistic primary root growth. Indeed, such strictly cell-autonomous mechanisms will logically lead to groups of (nearly) synchronously growing cells. Cell packages derived from each division of the initial cells will behave in a similar way irrespective of their position along the growth axis and produce growth zones changing periodically in size (when such a package leaves the DZ and enters the EZ). Even taking into account the inherent noise in cell behaviour, fixed developmental zones and smooth transitions in cell lengths are not feasible based on this type of regulation. On the other hand it cannot be excluded that some indirect mechanisms exist by which cells can circumvent the need for a spatial signal by deriving spatial information in an autonomous way. An example of this hypothesis could be the gravity-sensing columella cells that can extract spatial information through statholits in the root gravitropic response as modelled in [15].

Considering the inherent limitations of strictly cell-autonomous mechanisms and given the extensive evidence on the importance of phytohormones, it seems unlikely that those mechanisms are the main determinants of developmental zonation in the *Arabidopsis* root. We have demonstrated that spatial signals (for instance stemming from a biochemical gradient) can be a direct and (based on the visual and kinematic comparison) effective manner of instructing morphogenesis. This has been shown for various other life forms like Arthropoda and Vertebrata [63], [47], [37]. Our simulations regarding the effectiveness of auxin as the primary signal controlling root growth show that based on local auxin production a stable auxin pattern can be produced, however this potentially a slow process. A constant production per cell seems the most effective to this end. A faster breakdown [12] or efflux rate might aid in faster convergence. In any case, with an external auxin source such a pattern gradually fades out through growth dilution. Polar transport results in a lateral (radial) concentration gradient which conflicts with the ‘ULSR’. In fact our model does not even capture the extra volumetric dilution of auxin from the inner to the outer layers in three dimensions. Similar patterns were obtained by other studies like Grieneisen *et al.* [12] and Santuari *et al.* ([18]; their model did also not include the apoplastic compartment) and supported by various reporter studies (*e.g.* [64], [45]). Although Grieneisen *et al.* [12] have simulated stable (2D) growth of the root tip, the lateral gradients were not recognized as problematic probably due to the fact that relative movement of cells (cell sliding) is possible with their (cellular Potts instead of vertex-based) framework. We have proposed various strategies to circumvent the ULSR conflict. It has been pointed out that several hormones are exerting their regulatory effect on the root in a cell-layer specific way [9], [14]. This provides a way out of the ‘ULSR’ conundrum if accompanied by rapid transversal transmission to the other tissue layers. Candidate molecules to act as secondary transported signal are only just surfacing. On the other hand, even layer-driven growth by direct mechanical transduction was successful in producing a realistic root phenotype according to the three defined criteria. The role of auxin must by all means be understood in the complex context of various downstream response factors (with variations in levels, localisation, *etc.* (*e.g.* [48], [18]) and also of other hormones that interfere via their respective signalling pathway components. We constructed a model based on the antagonistic role of auxin and cytokinin in root development, with the SHY2 transcription factor as a central regulator of meristem size [65]–[67], [53], [54] and gibberellin (GA) dilution determining cell maturation. Simulations with this model were in accordance with the ULSR and reproduced visual and kinematic observations as well as the expected increase and decrease of meristem size upon addition of auxin and cytokinin [34]. By down-regulating PIN-mediated transport through the transcription factor SHY2, cytokinin effectively flattens the lateral auxin gradient, at the basal end of the division zone (‘transition zone’ [6]), thereby signalling the exit of proliferation and start of differentiation without conflicting the ULSR. GA dilution has been proposed before as part of a more intricate mechanism (including cell compartments and DELLA proteins, [19]) determining the exit of the elongation phase in a single cell row of the *Arabidopsis* root. We have used growth dilution of GA in a simplified form with a GA production term proportional to (initial) cell width to account for layer-specific differences in GA concentration. Cell elongation is defined to stop as soon as the GA concentration drops below a specific GA minimum, which depends on reaching a certain cell size (and accordingly length). This could account for the similarity in cell lengths at the end of the EZ which has been observed under different conditions in the *Arabidopsis* root [34].

Various definitions exist for the term ‘robustness’ [68]. We define it here in a broad sense as a property that allows a system to maintain its functions in the context of internal and external perturbations [69]. ‘Function’ pertains to maintaining stable growth upon internal changes or changing growth conditions (external). Some of our simulations showed that, in the absence of feedback from spatial cues, and driven by cell proliferation, cellular defects (for instance leading to differences in cell cycle time) can propagate. In contrast to cell-autonomous regulation a more dictatorial or hierarchical form of regulation (in this case via organ-scale spatial signals), is more robust to local perturbations. A possible trade-off of such a dictatorial system might be its sensitivity to mutations in the controlling network itself [70] as supported by experimental studies on PIN mutants [71], [72]. At the same time, such a high sensitivity to modulations in the control system may be beneficial for plants, being sedentary and therefore requiring a great deal of developmental plasticity. This trade-off also applies to the degree of dependence of cell growth and division. In theory, a completely independent regulation of these processes allows for a much wider morphological space to be accessible. Evidently, reaching stable balanced growth would become far more challenging. As the mechanistic coupling of growth and division is still poorly characterized [73], we opted here to couple those processes to various degrees in an artificial way, for instance as synchronized counters and timers, or concurring spatial transitions. Integrating an explicit model of the cell cycle in our models would undoubtedly further progress our understanding of how the coordination of those processes affects root morphogenesis.

Starting from a simple grid and by stepwise evaluation of regulatory mechanisms for cell division and expansion, we have obtained a model that produces realistic root growth according to well-defined criteria. In that respect spatial regulation appears to be essential, presumably with auxin as the central signal that is modulated by feedback interaction with cytokinin. This represents an important step towards predicting the *Arabidopsis* root phenotype under various conditions using vertex-based modelling. As indicated above, more advanced representations of cell wall mechanics, cell cycle regulation, *etc.* would benefit this model, and are currently being developed.

## Methods

A vertex-based modelling approach based on the modelling software VirtualLeaf [25] was used to represent a growing root apex that consists of nodes (vertices) connected by edges. Cells are polygons and cell wall segments correspond to the edges. Mechanically the edges are acting as linear springs which are opposing the tendency of cells to increase their area by virtue of their target areas. This is mathematically expressed as terms in a generalized energy function (or ‘Hamiltonian’), which was slightly modified to represent balanced linear growth (in the sense that small cells contribute equally to the energy function as larger cells, if they have identical relative growth rates).

Original Hamiltonian:
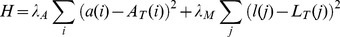
(1)Where indices *i* and *j* sum over all cells and polygon edges, respectively, *λ_A_* is a parameter setting the cells' resistance to compression or expansion, and *λ_M_* is a spring constant. *A_T_* is the cell's target area, *L_T_* the wall element target length.

In the new Hamiltonian the first term is replaced by:
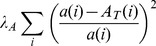
(2)The cell mechanics algorithm in VirtualLeaf randomly displaces all nodes to steer the system towards a global energy minimum. During this Metropolis Monte Carlo simulation any node displacement that leads to an energy decrease (or a small energy increase corresponding to a high Boltzmann probability) is accepted [25]. Added example input xml files contain the mechanical and numerical integration settings for simulation and illustrate the data structure used to describe the state of the cellular grid (Dataset S1). Besides the mechanical framework cells and cell walls are endowed with biochemical properties represented by reaction and transport equations and logical rules that determine when and how cell division occurs and how the cellular surface energy terms change. Our approach is to alter the structure of those equations and rules rather than just change parameter values to compare the output of the model versions.

The cellular organisation of the root tip was inspired by an earlier study [12] and always starts from a regular grid of rectangular cells, sometimes with narrower inner cell layers roughly in accordance to root anatomical characteristics [7]. The upper cell row always consisted of fixed nodes to enforce downward growth. Across all simulations the three lowest cell rows are not driven to grow in size (via their fixed target areas) and are meant to represent the complex of columella cells together with the quiescent centre (QC). Although this part of the root apex (root cap) has its own detailed developmental characteristics, we strictly focus here on the proximal/basal side of the QC while excluding the distal meristem as part of the growth process. The formative asymmetric divisions of the stem cells surrounding the QC [74] were neither included.

The models in principle describe growth in two dimensions but it was assumed that transversal expansion is severely constrained, which is in correspondence with experimental data indicating little or no tangential expansion (<1% h^−1^: [75]).

Cell walls behave as elastic springs with vertically oriented walls contributing less to the energy function (typically scaled down 5 or 10 times). This is sufficient to allow fluent elongation of cells along the root axis while keeping cell shape adequately constrained as required by turgor pressure acting on the cell walls. The output repertoire is limited both by our *a priori* model assumptions and by computational limits compared to what can be observed *in vitro* or *in vivo*. Briefly, lateral root formation and tropic responses such as gravitropism, coiling, waving, skewing are not produced. Model simulations do allow assessment of root growth rate and size, as well as detailed kinematics (the latter is in principle even more flexible than in a classical experimental setting where sampling has temporal and spatial limitations. It is also possible to assess deformation of overall organ shape to some degree (given the strong horizontal constraints) but especially defects in internal tissue organisation. At the cellular level the resolution of the simulations is sufficient to observe cell shape deformations.

The experimental data for comparison of simulated cell length distributions with the real *Arabidopsis* primary root (Figure 2) were obtained by kinematic analysis of one typical seedling grown in the same conditions as described in Beemster and Baskin [5]. Curve fitting was in general obtained by smoothing and interpolation at an interval of 5 to 25 µm using the kernel smoothing function ‘locpoly’ of the KernSmooth package [76] for the R statistical package (R foundation for Statistical Computing).

Table S1 presents a detailed overview of the models described in this paper. Except for *Model 9* and *Model 12* (*cf.* Figure 6 and 9), all models started off with 2 divisions of the cells in the row above the ‘QC’ (fourth row from below Figure 1B) to reduce the initial size of the cells and in some cases to introduce some asymmetry (noise) in that cell row. For cells to enter a new developmental phase requires the definition of a relative starting point in time. We have modelled this by associating a clock to each cell which is reset to zero as long as the daughter cells after cell division remain in contact with the non-growing cap structure. This results in cells in contact to the QC (hypothetically sensed by paracrine signalling for instance) remaining part of the stem cell niche ( = capable of self-renewal, ‘stemness’ [77]) and dividing at a pre-set frequency. Upon loss of contact with the QC-cap structure, the cells assume the identity of a typical meristematic (DZ) cell until eventually losing the potential for division after for instance a fixed time or number of divisions.

Cell-autonomous timer, counter, and sizer mechanisms were implemented similarly to the exemplary fragments of VirtualLeaf (pseudo-)code in Text S1. Pseudo-code fragments are also presented for the final *Model 12* in Text S1.

### Visualisation

In several instances visualization of simulation output consisted of colouring the cells according to either:

‘AS’ - Areal strain (rate):

To obtain a simple, approximate measure of the local expansion of the cells, the change of the area relative to the area in the previous simulation step was accounted for.
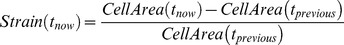
(3)


Red colouring typically indicates a positive strain rate and blue colouring a negative strain rate.

Or:

‘GP’ - Growth potential:

The ratio 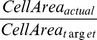 was used to colour the cellular grids in specific instances as a measure of the cellular growth potential (broadly interpreted as the ‘turgor pressure’). Red colouring indicates an energy driving areal growth, blue colouring an energy growth driving cell shrinking.

Or:

‘AU’ - Arbitrary concentration Units, with red to yellow colouring indicating increasing concentration values.

## Supporting Information

Dataset S1 **Model code.** All changed (w.r.t. the public VirtualLeaf code on http://virtualleaf.googlecode.com) C++ class files (.h for header and.cpp for implementation files): Cell.h, Cell.cpp, CellBase.h, CellBase.cpp, Mesh.h, Mesh.cpp, WallBase.h, WallBase.cpp, Wortel.h, and Wortel.cpp. The last one (the model ‘plugin’) contains a detailed and structured collection of code blocks for all 12 models used in this study. Moreover, input xml files are also needed for model simulation and 3 versions are supplied: Wortel(1).xml (for models 1–8,10,11), Wortel(2).xml (for model 9), and Wortel(3).xml (for model 12). They specify the typical parameter values for the numerical integration and Monte Carlo methods, input/output behaviour, as well as some kinetic constants in the header of the file. This is followed by the list of nodes, cells and walls of the cellular grid including their geometrical and biochemical attributes.
(ZIP)

Figure S1 **Similar output for counter- and timer-based models.** (A) Simulation output of *Model 3* (Table S1) with the exit from proliferation defined by a counter mechanism. The imposed growth and division rules have resulted in a highly regular grid with distinct zones of similar cell length. (B) Simulation output of the timer-based *Model 2* (but here without any noise added to the starting divisions of the tissue). This yields a very similar grid as in (A) at 99 h simulation time (the small differences are due to a few nodes in close proximity that have not collapsed due to the stochastic character of the Monte Carlo mechanical framework).
(TIF)

Figure S2 **Dynamic cell length distribution in a cell-autonomous model.** Cell length distribution at different time steps of *Model 2* (Table S1, Figure 3A–C). The distinct subpopulation of accelerating cells increases in length over time (*cf.* arrows: blue line around length 30 µm shifting to around length 60 µm in cyan), eventually adding to the ‘mature’ pool around length 120 µm as seen for the red line. At the last time step a new population of cells is ready to start accelerating growth.
(TIF)

Figure S3 **Influence of noise on cell-autonomous regulation.** (A) Plot equivalent to Figure 3B with noise added to individual cell cycle times (*Model 4* - Table S1, see also Figure 4A). Note the smoothened curve. The ‘*’ indicates from where steady growth starts. (B) Output of *Model 7* (Table S1). Upon release from the QC cells undergo 3 divisions based on reaching a cell layer-specific size (‘sizer’). As for other strictly cell-autonomous mechanisms, cells belong to groups of similarly sized and synchronously growing cells. Cell division is less synchronized which leads to a smoothened increase in cell numbers. (C) Cell length along the growth axis at time step 91.5 h shows broader cell length distributions (blue dots) when noise is added (*Model 4*, Table S1) compared to the red dots produced with *Model 2* (Table S1, same data as in Figure 3C).
(TIF)

Figure S4 **Spatial profiles of strain rate and longitudinal velocity based on non-cell-autonomous regulation.** (A) Approximate (fractional) longitudinal strain rates derived from the change in cell lengths (at 50 h and 55 h) obtained during the simulation of *Model 8* (*cf.* Figure 5C). Starting from the QC, values increase abruptly at the transition to the EZ. (B) Strain rates as in (A), however in this case data from 10 simulations with a different random number seed for the Monte Carlo sampling of the node positions were binned in 25 µm intervals and averaged (error bars indicating the degree of dispersion via the standard deviation). (C) In accordance with the strain rate profile (A) (which is the derivate of the velocity profile), the spatial profile of the longitudinal velocity at 50 h simulation time shows a sharp transition at around 200 µm from the QC (indicated by ‘*’). Data were derived from changes in cell positions over time (at 50 h and 55 h). (D) Velocity profile as in (C), but obtained through binning and averaging data from ten randomized simulations. The shape of the curve is smoother than in (C). The data points were fitted with a progress curve using the ‘polyloc’ method (*cf.* Methods).
(TIF)

Figure S5 **Temporal auxin patterns in a growing root.** Evolution of the total auxin levels (A,D,G), the total simulated root area (B,E,H), and the auxin concentrations (C,F,I), corresponding to Figure 6A–C. A strictly external source of auxin combined with local sinks results in saturation of the total auxin in the root (A), which together with a steady increase in surface area (B) leads to the auxin concentration converging to zero (C). A strictly internal auxin source (production rates proportional to cell areas) results in the total auxin level (D) increasing proportionally to the area increase (E), and the auxin concentration slowly converging to a steady state (F). A strictly internal auxin source (production rates constant per cell) results in similar patterns as for (D–F) with a steady state in auxin concentration (I) as a function of the total auxin level (G) and area (H).
(TIF)

Figure S6
**Effect of parameter variations on spatial auxin patterns I.** Auxin concentration versus distance from the base of a simulated root for different cell files (2nd, 3rd, 5th from left) in a static mesh with variations in the diffusion constant D and the PIN exporter rate constant 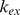. The corresponding tissues with their auxin concentration pattern were included (yellow colouring; arbitrary units: AUs). (A) D = 1000 µm^2^/min, 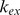 = 1200 µm/min; (B) D = 6000 µm^2^/min, 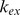 = 1200 µm/min; (C) D = 36000 µm^2^/min, 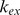 = 1200 µm/min; (D) D = 1000 µm^2^/min, 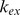 = 7200 µm/min; (E) D = 6000 µm^2^/min, 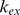 = 7200 µm/min; (F) D = 36000 µm^2^/min, 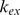 = 7200 µm/min. Increasing D (compare A–C and D–F) flattens the auxin gradient, whereas increasing 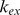 sharpens the gradient. Note that similar ratios of 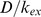 (such as in A and E, and B and F) lead to similar output. Fixed parameter values: 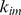 = 600 µm/min, local auxin production 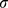 = 10 AU/min, net auxin flux of top row = 10^6^/min, apoplast thickness = 1 µm. As in Figure 6D a transversal gradient of auxin is visible.(TIF)

Figure S7
**Effect of parameter variations on spatial auxin patterns II.** Auxin concentration versus distance from the base of a simulated root for different cell files (2nd, 3rd, 5th from left) in a static mesh with variations in the importer rate constant 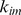. (A) 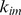 = 100 µm/min; (B) 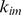 = 600 µm/min; (C) 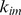 = 3600 µm/min. The other parameters are the same as in Fig. S6B. Increasing 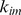 to sufficiently high values, as with the diffusion coefficient D, flattens the auxin gradient.(TIF)

Figure S8
**Effect of parameter variations on spatial auxin patterns III.** Auxin concentration versus distance from the base of a simulated root for different cell files (2nd, 3rd, 5th from left) in a static mesh with variations in the local production rate 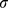. (A) 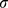 = 2 µm/min; (B) 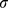 = 10 µm/min; (C) 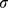 = 50 µm/min. The other parameters are the same as in Fig. S6B. Increasing 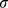 to sufficiently high values amplifies the overall auxin gradient.(TIF)

Figure S9 **Effect of parameter variations on spatial auxin patterns IV.** Auxin concentration versus distance from the base of a simulated root for different cell files (2nd, 3rd, 5th from left) in a static mesh with variations in the net total auxin influx of the top row of cells *F′*. (A) *F′* = 2.10^5^ 1/min; (B) *F′* = 10^6^ 1/min; (C) *F′* = 5.10^6^ 1/min. The other parameters are the same as in Fig. S6B. Increasing *F′* to sufficiently high values amplifies the overall auxin gradient.
(TIF)

Figure S10 ***Model 12*** **leads to stable root growth with smooth transition in cell length obtained through SHY2 action.** Simulation output demonstrates various aspects of *Model 12*. (A) Chemical concentration profiles (after 10 h simulation) across the 8^th^ cell file (from the left) of SHY2 (red, AUs) and auxin (yellow, log^10^ values as dimensions). For auxin the profile is also plotted for the first cell file (from the left, coloured green, log^10^ values) to demonstrate the flattening effect of SHY2 on the auxin gradient. (B) Plot of root length versus simulation time showing steady linear organ growth after approximately 9 hours. (C) Plot depicting the cell length along the principal growth axis (at 60 h). Note that cell lengths vary smoothly from DZ to EZ (some synchronicity for cells at a similar axial position is visible, since no noise was added to the first divisions contrary to simulations pictured in for instance *Fig. 5*).
(TIF)

Figure S11
**Effect of parameter variations on spatial auxin patterning in** ***Model 12*** **.** The simulation output of *Model 12* (yellow colouring; arbitrary units: AU) is illustrated here for different parameter values of auxin diffusion (D) and a-polar transport (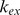). (A) D = 900 µm^2^/min, 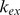 = 2000 µm/min; (B) D = 600 µm^2^/min, 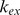 = 2000 µm/min; (C) D = 3600 µm^2^/min, 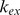 = 2000 µm/min; (D) D = 900 µm^2^/min, 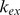 = 4000 µm/min. Increasing D (compare (B), (A), and (C)) expands the zone with high auxin activity and together with it the meristem, whereas increasing 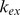 (compare (D), (A), and (E)) has the opposite effect. Note that keeping the 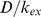
constant (*cf.* Figure S6) should lead to similar output. Fixed parameter values were as in Table S2.(TIF)

Figure S12
***Model 12*** **is robust to small parameter variations.** The simulation output of *Model 12* (yellow colouring; arbitrary units: AU) is shown here for a 10% increase of different parameter values related to hormone transport: (A) simulation based on the reference parameter set (Table S2); (B) D[0] perturbed; (C) 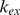 perturbed; (D) 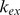 perturbed; (E) 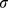 perturbed; (F) D[1] perturbed. The output is highly similar, which is also the case if these parameter values are decreased by 10% (results not shown), demonstrating local robustness/stability of the simulated output to changes of these parameters.(TIF)

Table S1 **Model overview.** Overview of the models used in this study. Various categories w.r.t. developmental decisions are presented. Column (3) specifies the transition between division and elongation zone (DZ and EZ, respectively) with in parentheses the number of division or time since release form the QC; column (4) specifies the transition to mature (differentiated) cells based on timing since the release from the QC or a spatial signal at a fixed distance from the root apex; column (5) specifies whether division rate is determined via a timer or sizer mechanism; and column (6) how cellular growth rates are defined. Developmental events can be determined to happen after a fixed duration (‘Timer’), a fixed number of divisions (‘Counter’), a fixed cell size (‘Sizer’), and a fixed distance from the root apex (‘Positional’, *i.e.* a ruler). For Models 10–12 more complicated regulatory mechanisms are specified. In Model 4 extra random noise was added to the timer (+/− max. 25%). For Model 5 cell division is dependent on a timer mechanism, with a different cell cycle time for inner and outer layers. Model 6 uses a uniform size (area) criterion for cell division, irrespectively of geometry. For Model 7 the size criterion is adapted to the difference in the width between inner and outer layers, thereby acting effectively as a length criterion. In the other (non-cell autonomous) cases the cell division sizers differs between layers. It is usually assumed that DZ and EZ have a characteristic elongation rate (expressed as a constant addition to the target area or a fraction of the current cell area). Layer-specific differences are indicated (by column numbers) if necessary. All models are identical in that the columella/QC cells (lower 3 cell rows) cannot divide. For timer and counter based models the contact with the QC cells is important to keep them dividing. As soon as they lose contact the respective counter or timer starts to increase. For non-cell-autonomous models a morphogen threshold determines if cells keep dividing. For each model cell division occurs through the insertion of a new horizontal wall dividing the cell in half. For models using hormone transport parameter values were used in accordance with known modelling studies such as [12], [17], and Wabnik *et al.*, 2010 [79]. In general reaction and transport equations are discussed in Text S1. A detailed parameter description can be found in the respective figures or in Table S2.
(DOCX)

Table S2 **Overview of kinetic parameters of** ***Model 12*** **.** For *Model 12* the auxin reaction and transport parameters D[0], k_export, and k_import, were adjusted to produce an auxin gradient with a more pronounced and central maximum such as found in various reporter studies (see for instance Fig. 3c in [12] and Fig. 1A in [18]). Figures S11 and S12 illustrate parameter dependent behaviour and sensitivity. The model behaviour is robust to hormone transport parameters changes and even larger changes can in principle be accommodated based on the balance between a-polar and polar auxin transport and stable negative feedback regulation of auxin and cytokinin.
(DOCX)

Text S1 **Background information on model implementation.**
(DOCX)

Video S1 **Realistic root growth through non-cell-autonomous regulation.** Movie obtained through simulation of *Model 8* (Table S1) for 84 hours shows that steady growth is reached after a transient time (of roughly 30 hours). Colouring is in accordance with areal strain rates (‘AS’, *cf.* Methods) and shows that the EZ, which is typically more intensely coloured, reaches a stable size, while moving down.
(MP4)

## References

[1] Food and Agriculture Organization of the United Nations (2013) The State of Food Insecurity in the World 2013. Available: http://www.fao.org/docrep/018/i3434e/i3434e00.htm

[2] Petricka JJ, Winter CM, Benfey PN (2012) Control of Arabidopsis root development. Annu Rev Plant Biol 63: 563–590. 22404466 10.1146/annurev-arplant-042811-105501 PMC3646660

[3] Fiorani F, Beemster GT (2006) Quantitative analyses of cell division in plants. Plant Mol Biol 60: 963–979. 16724264 10.1007/s11103-005-4065-2

[4] Nelissen H, Rymen B, Coppens F, Dhondt S, Fiorani F, et al (2013) Kinematic analysis of cell division in leaves of mono- and dicotyledonous species: a basis for understanding growth and developing refined molecular sampling strategies. Methods Mol Biol 959: 247–264. 23299681 10.1007/978-1-62703-221-6_17

[5] Beemster GT, Baskin TI (1998) Analysis of cell division and elongation underlying the developmental acceleration of root growth in Arabidopsis thaliana. Plant Physiol 116: 1515–1526. 9536070 10.1104/pp.116.4.1515 PMC35060

[6] Verbelen J-P, De Cnodder T, Le J, Vissenberg K, Baluška F (2006) Root apex of Arabidopsis thaliana consists of four distinct zones of growth activities: meristematic zone, transition zone, fast elongation zone, and growth terminating zone. Plant Signal and Behav 1: 296–304. 10.4161/psb.1.6.3511 PMC2634244 19517000

[7] Dolan L, Janmaat K, Willemsen V, Linstead P, Poethig S, et al (1993) Cellular organisation of the Arabidopsis thaliana root. Development 119: 71–84. 8275865 10.1242/dev.119.1.71

[8] Paciorek T, Bergmann DC (2010) The secret to life is being different: asymmetric divisions in plant development. Curr Opin Plant Biol 13: 661–669. 20970370 10.1016/j.pbi.2010.09.016

[9] Ubeda-Tomás S, Beemster GT, Bennett MJ (2012) Hormonal regulation of root growth: integrating local activities into global behaviour. Trends Plant Sci 17: 326–331. 22401844 10.1016/j.tplants.2012.02.002

[10] De Vos D, Dzhurakhalov A, Draelants D, Bogaerts I, Kalve S, et al (2012) Towards mechanistic models of plant organ growth. J Exp Bot 63: 3325–3337. 22371079 10.1093/jxb/ers037

[11] Daetwyler HD, Calus MP, Pong-Wong R, de Los Campos G, Hickey JM (2013) Genomic prediction in animals and plants: simulation of data, validation, reporting, and benchmarking. Genetics 193: 347–365. 23222650 10.1534/genetics.112.147983 PMC3567728

[12] Grieneisen VA, Xu J, Marée AF, Hogeweg P, Scheres B (2007) Auxin transport is sufficient to generate a maximum and gradient guiding root growth. Nature 449: 1008–1013. 17960234 10.1038/nature06215

[13] Laskowski M, Grieneisen VA, Hofhuis H, Hove CA, Hogeweg P, et al (2008) Root system architecture from coupling cell shape to auxin transport. PLoS Biol 6: e307. 19090618 10.1371/journal.pbio.0060307 PMC2602721

[14] Swarup R, Kramer EM, Perry P, Knox K, Leyser HM, et al (2005) Root gravitropism requires lateral root cap and epidermal cells for transport and response to a mobile auxin signal. Nat Cell Biol 7: 1057–1065. 16244669 10.1038/ncb1316

[15] Band LR, Wells DM, Larrieu A, Sun J, Middleton AM, et al (2012) Root gravitropism is regulated by a transient lateral auxin gradient controlled by a tipping-point mechanism. Proc Natl Acad Sci U S A 109: 4668–4673. 22393022 10.1073/pnas.1201498109 PMC3311388

[16] Mironova VV, Omelyanchuk NA, Yosiphon G, Fadeev SI, Kolchanov NA, et al (2010) A plausible mechanism for auxin patterning along the developing root. BMC Syst Biol 4: 98. 20663170 10.1186/1752-0509-4-98 PMC2921385

[17] Cruz-Ramírez A, Díaz-Triviño S, Blilou I, Grieneisen VA, Sozzani R, et al (2012) A bistable circuit involving SCARECROW-RETINOBLASTOMA integrates cues to inform asymmetric stem cell division. Cell 150: 1002–1015. 22921914 10.1016/j.cell.2012.07.017 PMC3500399

[18] Santuari L, Scacchi E, Rodriguez-Villalon A, Salinas P, Dohmann EM, et al (2011) Positional information by differential endocytosis splits auxin response to drive Arabidopsis root meristem growth. Curr Biol 21: 1918–1923. 22079112 10.1016/j.cub.2011.10.002

[19] Band LR, Úbeda-Tomás S, Dyson RJ, Middleton AM, Hodgman TC, et al (2012) Growth-induced hormone dilution can explain the dynamics of plant root cell elongation. Proc Natl Acad Sci U S A 109: 7577–7582. 22523244 10.1073/pnas.1113632109 PMC3358831

[20] Erickson RO (1986) Symplastic growth and symplasmic transport. Plant Physiol 82: 1153. 16665152 10.1104/pp.82.4.1153 PMC1056276

[21] Fozard JA, Lucas M, King JR, Jensen OE (2013) Vertex-element models for anisotropic growth of elongated plant organs. Front Plant Sci 4: 233. 23847638 10.3389/fpls.2013.00233 PMC3706750

[22] Uyttewaal M, Burian A, Alim K, Landrein B, Borowska-Wykręt D, et al (2012) Mechanical stress acts via katanin to amplify differences in growth rate between adjacent cells in Arabidopsis. Cell 149: 439–451. 22500806 10.1016/j.cell.2012.02.048

[23] Besnard F, Vernoux T, Hamant O (2011) Organogenesis from stem cells in planta: multiple feedback loops integrating molecular and mechanical signals. Cell Mol Life Sci 68: 2885–2906. 21655916 10.1007/s00018-011-0732-4 PMC11115100

[24] Hamant O, Traas J (2010) The mechanics behind plant development. New Phytol 185: 369–385. 20002316 10.1111/j.1469-8137.2009.03100.x

[25] Merks RM, Guravage M, Inzé D, Beemster GT (2011) VirtualLeaf: an open-source framework for cell-based modeling of plant tissue growth and development. Plant Physiol 155: 656–666. 21148415 10.1104/pp.110.167619 PMC3032457

[26] Green PB (1976) Growth and Cell Pattern Formation on an Axis: Critique of Concepts, Terminology, and Modes of Study. Botanical Gazette 137: 187–202.

[27] Jacobs T (1997) Why Do Plant Cells Divide? Plant Cell 9: 1021–1029. 12237374 10.1105/tpc.9.7.1021 PMC156976

[28] Silk WK, Erickson RO (1979) Kinematics of plant growth. J Theor Biol 76: 481–501. 439916 10.1016/0022-5193(79)90014-6

[29] Beemster GT, Fiorani F, Inzé D (2003) Cell cycle: the key to plant growth control? Trends Plant Sci 8: 154–158. 12711226 10.1016/S1360-1385(03)00046-3

[30] De Veylder L, Beeckman T, Inzé D (2007) The ins and outs of the plant cell cycle. Nat Rev Mol Cell Biol 8: 655–665. 17643126 10.1038/nrm2227

[31] Tsukaya H (2002) Interpretation of mutants in leaf morphology: genetic evidence for a compensatory system in leaf morphogenesis that provides a new link between cell and organismal theories. Int Rev Cytol 217: 1–39. 12019561 10.1016/s0074-7696(02)17011-2

[32] Ivanov VB, Dobrochaev AE, Baskin TI (2002) What the Distribution of Cell Lengths in the Root Meristem Does and Does Not Reveal About Cell Division. J Plant Growth Regul 21: 60–67. 11997812 10.1007/s003440010051

[33] Ivanov VB (1974) Cellular Basis of Plant Growth. Moscow: Nauka Press: 40–41.

[34] Beemster GT, Baskin TI (2000) Stunted plant 1 mediates effects of cytokinin, but not of auxin, on cell division and expansion in the root of Arabidopsis. Plant Physiol 124: 1718–1727. 11115888 10.1104/pp.124.4.1718 PMC59869

[35] González-Fernández A, López-Sáez JF, Moreno P, Giménez-Martín G (1968) A model for dynamics of cell division cycle in onion roots. Protoplasma 65: 263–276. 5685727 10.1007/BF01682532

[36] Temple S, Raff MC (1986) Clonal analysis of oligodendro- cyte development in culture: evidence for a developmen- tal clock that counts cell divisions. Cell 44: 773–779. 3948247 10.1016/0092-8674(86)90843-3

[37] Hester SD, Belmonte JM, Gens JS, Clendenon SG, Glazier JA (2011) A multi-cell, multi-scale model of vertebrate segmentation and somite formation. PLoS Comput Biol 7: e1002155. 21998560 10.1371/journal.pcbi.1002155 PMC3188485

[38] Qu Z, MacLellan WR, Weiss JN (2003) Dynamics of the cell cycle: checkpoints, sizers, and timers. Biophys J 85: 3600–3611. 14645053 10.1016/S0006-3495(03)74778-X PMC1303665

[39] Li B, Shao B, Yu C, Ouyang Q, Wang H (2010) A mathematical model for cell size control in fission yeast. J Theor Biol 264: 771–781. 20303984 10.1016/j.jtbi.2010.03.023

[40] Csikász-Nagy A, Battogtokh D, Chen KC, Novák B, Tyson JJ (2006) Analysis of a generic model of eukaryotic cell-cycle regulation. Biophys J 90: 4361–4379. 16581849 10.1529/biophysj.106.081240 PMC1471857

[41] Roeder AH (2012) When and where plant cells divide: a perspective from computational modeling. Curr Opin Plant Biol 15: 638–644. 22939706 10.1016/j.pbi.2012.08.002

[42] Silk WK, Hsiao TC, Diedenhofen U, Matson C (1986) Spatial Distributions of Potassium, Solutes, and Their Deposition Rates in the Growth Zone of the Primary Corn Root. Plant Physiol 82: 853–858. 16665121 10.1104/pp.82.3.853 PMC1056218

[43] Walter A, Spies H, Terjung S, Küsters R, Kirchgessner N, Schurr U (2002) Spatio-temporal dynamics of expansion growth in roots: automatic quantification of diurnal course and temperature response by digital image sequence processing. J Exp Botany 53: 689–698. 11886889 10.1093/jexbot/53.369.689

[44] van der Weele CM, Jiang HS, Palaniappan KK, Ivanov VB, Palaniappan K, et al (2003) A new algorithm for computational image analysis of deformable motion at high spatial and temporal resolution applied to root growth. Roughly uniform elongation in the meristem and also, after an abrupt acceleration, in the elongation zone. Plant Physiol 132: 1138–1148. 12857796 10.1104/pp.103.021345 PMC526269

[45] Brunoud G, Wells DM, Oliva M, Larrieu A, Mirabet V, et al (2012) A novel sensor to map auxin response and distribution at high spatio-temporal resolution. Nature 482: 103–106. 22246322 10.1038/nature10791

[46] Beeckman T, Burssens S, Inzé D (2001) The peri-cell-cycle in Arabidopsis. J Exp Bot 52: 403–411. 11326046 10.1093/jexbot/52.suppl_1.403

[47] Wartlick O, Mumcu P, Jülicher F, Gonzalez-Gaitan M (2011) Understanding morphogenetic growth control – lessons from flies. Nat Rev Mol Cell Biol 12: 594–604. 21850035 10.1038/nrm3169

[48] Vernoux T, Brunoud G, Farcot E, Morin V, Van den Daele H, et al (2011) The auxin signalling network translates dynamic input into robust patterning at the shoot apex. Mol Syst Biol 7: 508. 21734647 10.1038/msb.2011.39 PMC3167386

[49] Aloni R (1982) Role of cytokinin in differentiation of secondary xylem fibers. Plant Physiol 70: 1631–1633. 16662733 10.1104/pp.70.6.1631 PMC1065944

[50] Silverstone AL, Chang C, Krol E, Sun TP (1997) Developmental regulation of the gibberellin biosynthetic gene GA1 in Arabidopsis thaliana. Plant J 12: 9–19. 9263448 10.1046/j.1365-313x.1997.12010009.x

[51] Birnbaum K, Shasha DE, Wang JY, Jung JW, Lambert JM, et al (2003) A gene expression map of the Arabidopsis root. Science 302: 1956–1960. 14671301 10.1126/science.1090022

[52] Nawy T, Lee JY, Colinas J, Wang JY, Thongrod SC, et al (2005) Transcriptional profile of the Arabidopsis root quiescent center. Plant Cell 17: 1908–1925. 15937229 10.1105/tpc.105.031724 PMC1167541

[53] Moubayidin L, Di Mambro R, Sabatini S (2009) Cytokinin-auxin crosstalk. Trends Plant Sci 14: 557–562. 19734082 10.1016/j.tplants.2009.06.010

[54] Moubayidin L, Perilli S, Dello Ioio R, Di Mambro R, Costantino P, et al (2010) The rate of cell differentiation controls the Arabidopsis root meristem growth phase. Curr Biol 20: 1138–1143. 20605455 10.1016/j.cub.2010.05.035

[55] Muraro D, Byrne H, King J, Bennett M (2013) The role of auxin and cytokinin signalling in specifying the root architecture of Arabidopsis thaliana. J Theor Biol 317: 71–86. 23026765 10.1016/j.jtbi.2012.08.032

[56] Liu J, Mehdi S, Topping J, Tarkowski P, Lindsey K (2010) Modelling and experimental analysis of hormonal crosstalk in Arabidopsis. Mol Syst Biol 6: 373. 20531403 10.1038/msb.2010.26 PMC2913391

[57] Tyson JJ, Novak B (2008) Temporal organization of the cell cycle. Curr Biol 18: R759–R768. 18786381 10.1016/j.cub.2008.07.001 PMC2856080

[58] Elowitz MB, Leibler S (2000) A synthetic oscillatory network of transcriptional regulators. Nature 403: 335–338. 10659856 10.1038/35002125

[59] Stricker J, Cookson S, Bennett MR, Mather WH, Tsimring LS, et al (2008) A fast, robust and tunable synthetic gene oscillator. Nature 456: 516–519. 18971928 10.1038/nature07389 PMC6791529

[60] Harashima H, Dissmeyer N, Schnittger A (2013) Cell cycle control across the eukaryotic kingdom. Trends Cell Biol 23: 345–356. 23566594 10.1016/j.tcb.2013.03.002

[61] van Leeuwen F, Frederiks F, Terweij M, De Vos D, Bakker BM (2012) News about old histones: a role for histone age in controlling the epigenome. Cell Cycle 11: 11–12. 22157090 10.4161/cc.11.1.18779

[62] Counter CM (1996) The roles of telomeres and telomerase in cell life span. Mutat Res 366: 45–63. 8921986 10.1016/s0165-1110(96)90006-8

[63] Jaeger J (2011) The gap gene network. Cell Mol Life Sci 68: 243–274. 20927566 10.1007/s00018-010-0536-y PMC3016493

[64] Sabatini S, Beis D, Wolkenfelt H, Murfett J, Guilfoyle T, et al (1999) An auxin-dependent distal organizer of pattern and polarity in the Arabidopsis root. Cell 99: 463–472. 10589675 10.1016/s0092-8674(00)81535-4

[65] Dello Ioio R, Nakamura K, Moubayidin L, Perilli S, Taniguchi M, et al (2008) A genetic framework for the control of cell division and differentiation in the root meristem. Science 322: 1380–1384. 19039136 10.1126/science.1164147

[66] Dello Ioio R, Galinha C, Fletcher AG, Grigg SP, Molnar A, et al (2012) A PHABULOSA/cytokinin feedback loop controls root growth in Arabidopsis. Curr Biol 22: 1699–1704. 22902752 10.1016/j.cub.2012.07.005

[67] Muraro D, Byrne H, King J, Voss U, Kieber J, et al (2011) The influence of cytokinin-auxin cross-regulation on cell-fate determination in Arabidopsis thaliana root development. J Theor Biol 283: 152–167. 21640126 10.1016/j.jtbi.2011.05.011

[68] Kitano H (2007) Towards a theory of biological robustness. Mol Syst Biol 3: 137. 17882156 10.1038/msb4100179 PMC2013924

[69] Kitano H (2004) Biological robustness. Nat Rev Genet 5: 826–837. 15520792 10.1038/nrg1471

[70] Quinton-Tulloch MJ, Bruggeman FJ, Snoep JL, Westerhoff HV (2013) Trade-off of dynamic fragility but not of robustness in metabolic pathways in silico. FEBS J 280: 160–173. 23121761 10.1111/febs.12057

[71] Friml J, Vieten A, Sauer M, Weijers D, Schwarz H, et al (2003) Efflux-dependent auxin gradients establish the apical–basal axis of Arabidopsis. Nature 426: 147–153. 14614497 10.1038/nature02085

[72] Blilou I, Xu J, Wildwater M, Willemsen V, Paponov I, et al (2005) The PIN auxin efflux facilitator network controls growth and patterning in Arabidopsis roots. Nature 433: 39–44. 15635403 10.1038/nature03184

[73] Gonzalez N, Vanhaeren H, Inzé D (2012) Leaf size control: complex coordination of cell division and expansion. Trends Plant Sci 17: 332–340. 22401845 10.1016/j.tplants.2012.02.003

[74] Scheres B (2007) Stem-cell niches: Nursery rhymes across kingdoms. Nat Rev Mol Cell Biol 8: 345–354. 17450175 10.1038/nrm2164

[75] Baskin TI, Beemster GT, Judy-March JE, Marga F (2004) Disorganization of cortical microtubules stimulates tangential expansion and reduces the uniformity of cellulose microfibril alignment among cells in the root of Arabidopsis. Plant Physiol 135: 2279–2290. 15299138 10.1104/pp.104.040493 PMC520797

[76] Wand MP, Jones MC (1995) Kernel Smoothing. Chapman & Hall, London.

[77] van den Berg C, Willemsen V, Hendriks G, Weisbeek P, Scheres B (1997) Short-range control of cell differentiation in the Arabidopsis root meristem. Nature 390: 287–289. 9384380 10.1038/36856

[78] Ubeda-Tomás S, Federici F, Casimiro I, Beemster GT, Bhalerao R, et al (2009) Gibberellin signaling in the endodermis controls Arabidopsis root meristem size. Curr Biol 19: 1194–1199. 19576770 10.1016/j.cub.2009.06.023

[79] Wabnik K, Kleine-Vehn J, Balla J, Sauer M, Naramoto S, et al (2010) Emergence of tissue polarization from synergy of intracellular and extracellular auxin signaling. Mol Syst Biol 6: 447. 21179019 10.1038/msb.2010.103 PMC3018162

