# Supplementary figures and images for: Putting Theory to the Test: Which Regulatory Mechanisms Can Drive Realistic Growth of a Root?
Source: PLoS Comput Biol. 2014 Oct 30;10(10):e1003910. doi: 10.1371/journal.pcbi.1003910 (PMC4214622; doi:10.1371/journal.pcbi.1003910)

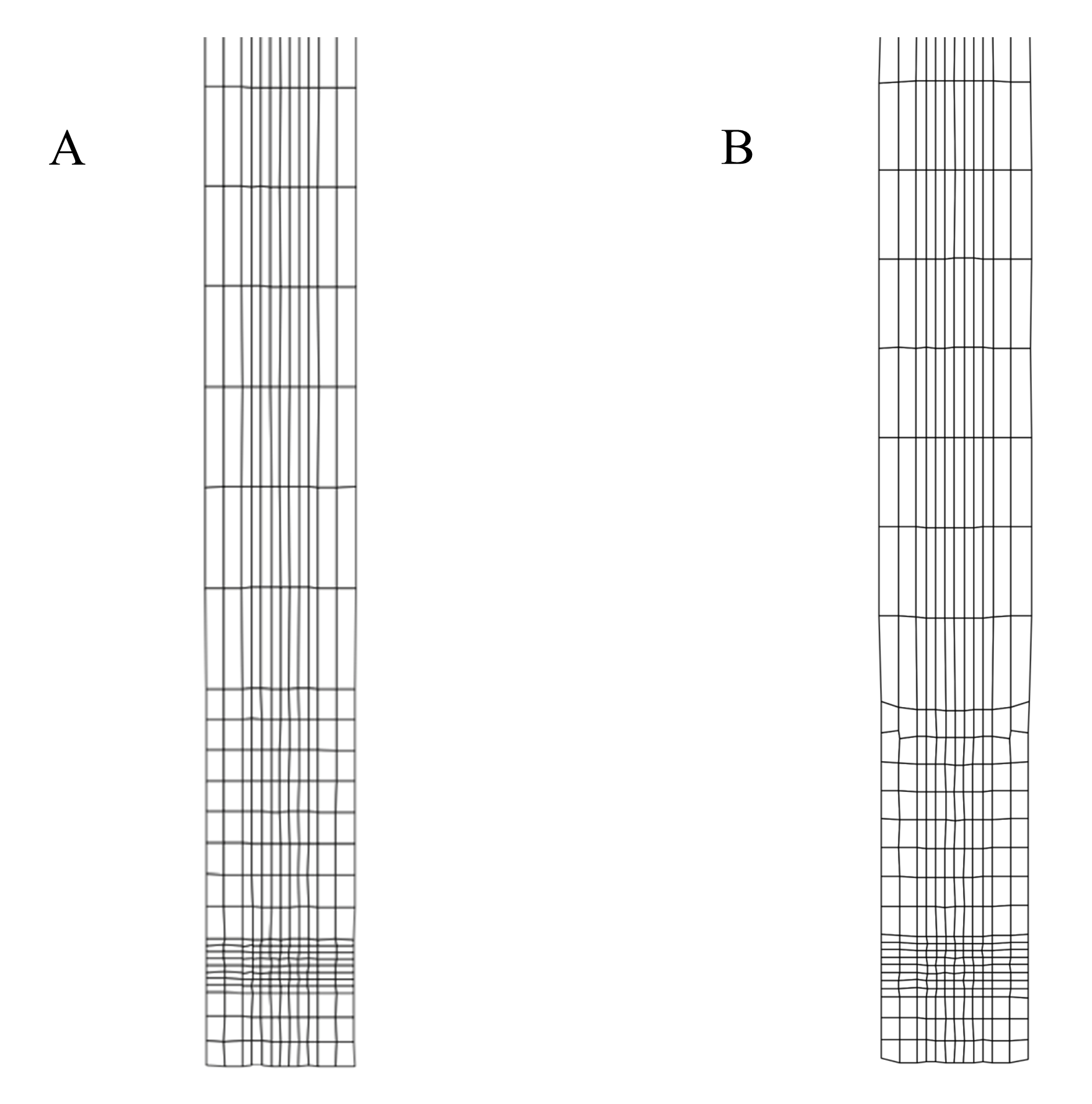

Supplement: Figure S1 — Similar output for counter- and timer-based models. (A) Simulation output of Model 3 (Table S1) with the exit from proliferation defined by a counter mechanism. The imposed growth and division rules have resulted in a highly regular grid with distinct zones of similar cell length. (B) Simulation output of the timer-based Model 2 (but here without any noise added to the starting divisions of the tissue). This yields a very similar grid as in (A) at 99 h simulation time (the small differences are due to a few nodes in close proximity that have not collapsed due to the stochastic character of the Monte Carlo mechanical framework). (TIF) [file pcbi.1003910.s002.tif]

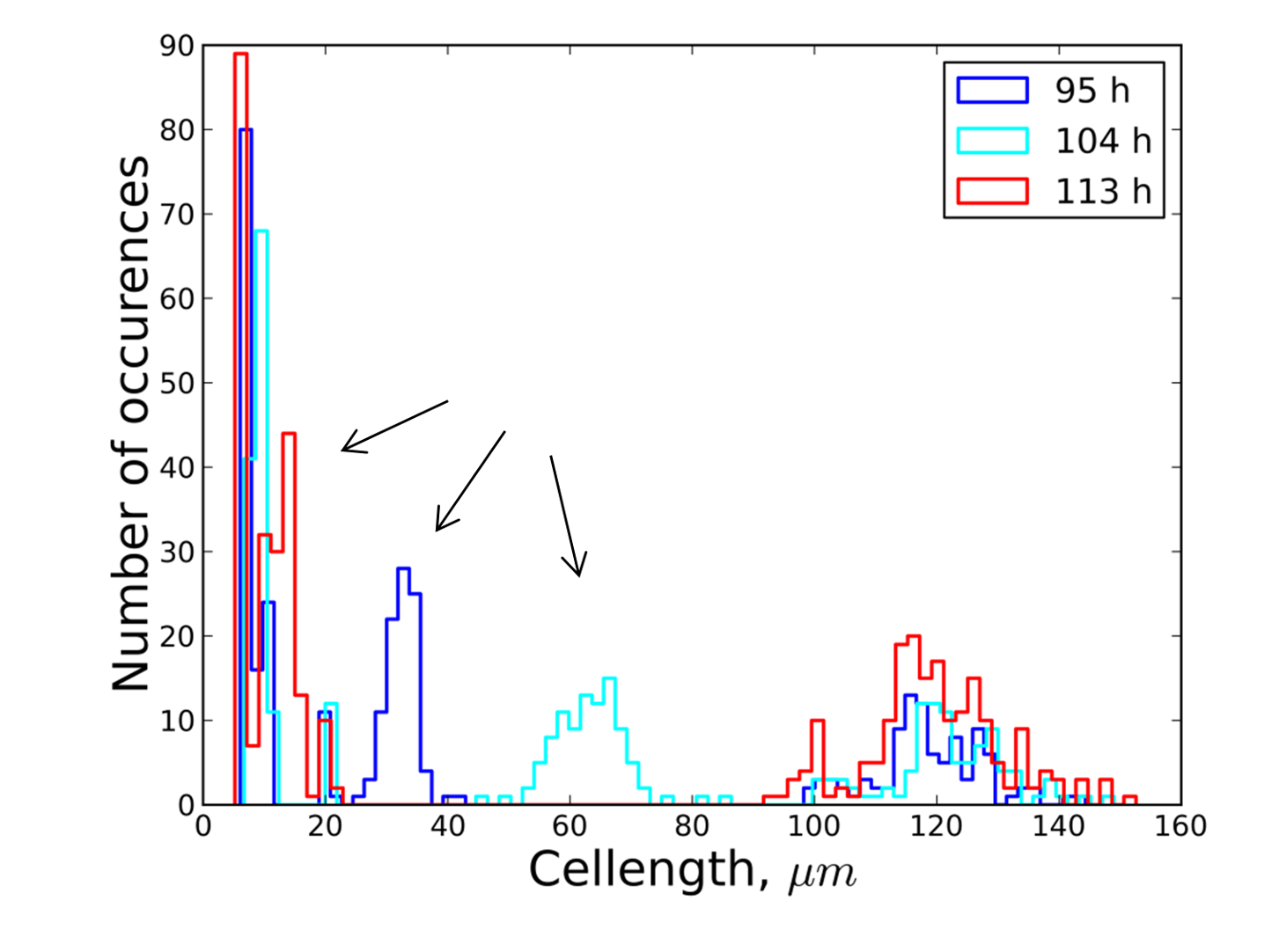

Supplement: Figure S2 — Dynamic cell length distribution in a cell-autonomous model. Cell length distribution at different time steps of Model 2 (Table S1, Figure 3A–C). The distinct subpopulation of accelerating cells increases in length over time (cf. arrows: blue line around length 30 µm shifting to around length 60 µm in cyan), eventually adding to the ‘mature’ pool around length 120 µm as seen for the red line. At the last time step a new population of cells is ready to start accelerating growth. (TIF) [file pcbi.1003910.s003.tif]

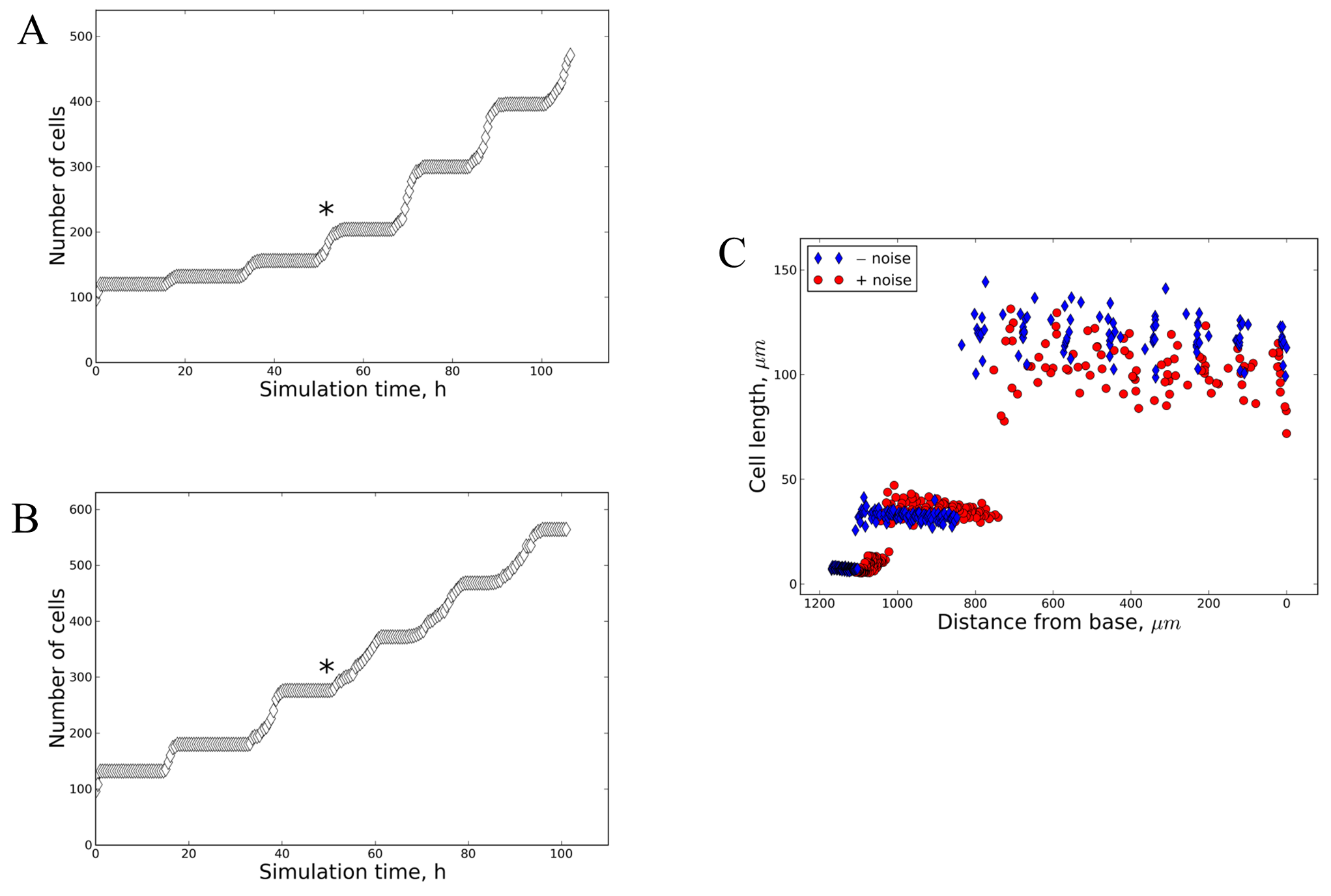

Supplement: Figure S3 — Influence of noise on cell-autonomous regulation. (A) Plot equivalent to Figure 3B with noise added to individual cell cycle times (Model 4 - Table S1, see also Figure 4A). Note the smoothened curve. The ‘*’ indicates from where steady growth starts. (B) Output of Model 7 (Table S1). Upon release from the QC cells undergo 3 divisions based on reaching a cell layer-specific size (‘sizer’). As for other strictly cell-autonomous mechanisms, cells belong to groups of similarly sized and synchronously growing cells. Cell division is less synchronized which leads to a smoothened increase in cell numbers. (C) Cell length along the growth axis at time step 91.5 h shows broader cell length distributions (blue dots) when noise is added (Model 4, Table S1) compared to the red dots produced with Model 2 (Table S1, same data as in Figure 3C). (TIF) [file pcbi.1003910.s004.tif]

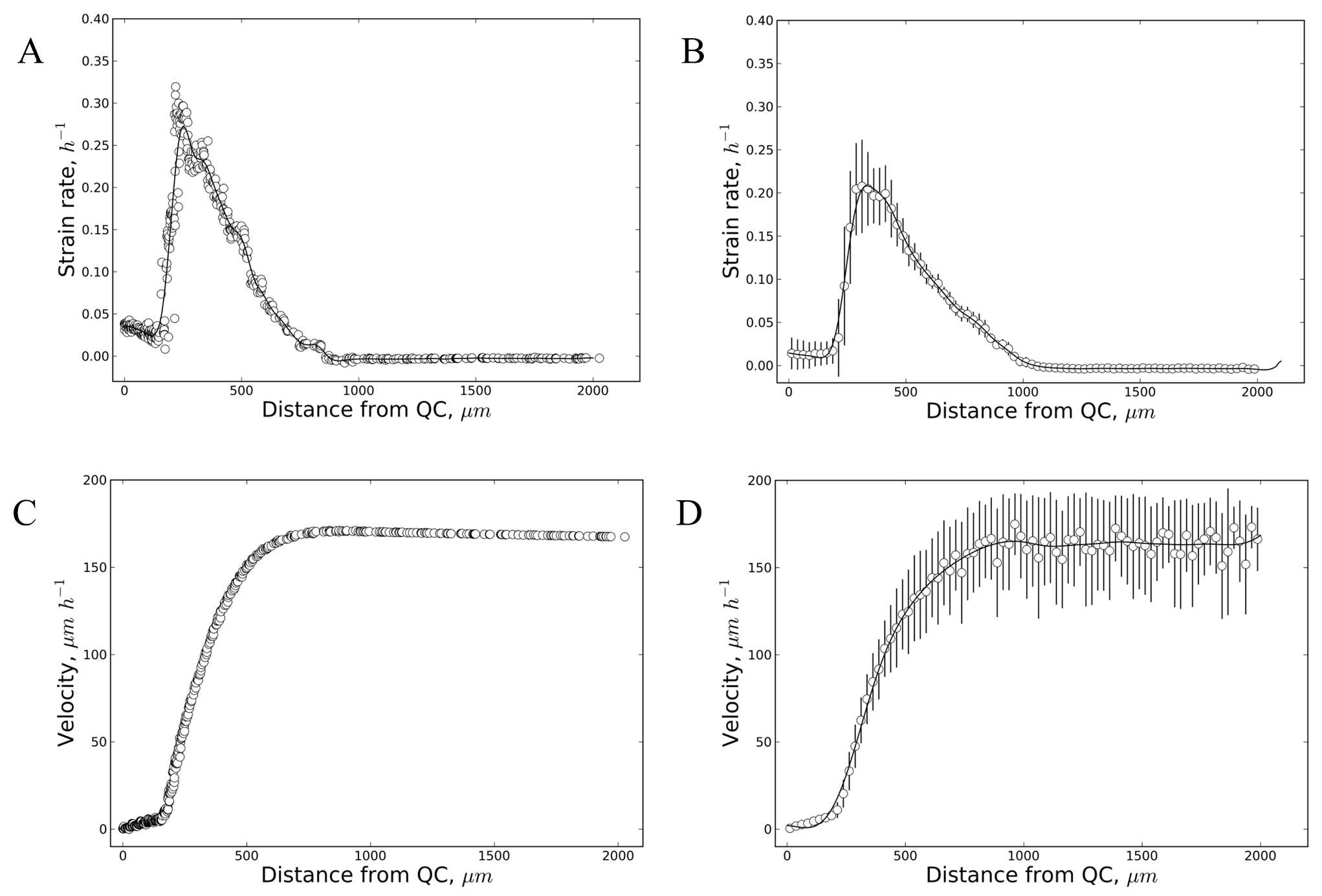

Supplement: Figure S4 — Spatial profiles of strain rate and longitudinal velocity based on non-cell-autonomous regulation. (A) Approximate (fractional) longitudinal strain rates derived from the change in cell lengths (at 50 h and 55 h) obtained during the simulation of Model 8 (cf. Figure 5C). Starting from the QC, values increase abruptly at the transition to the EZ. (B) Strain rates as in (A), however in this case data from 10 simulations with a different random number seed for the Monte Carlo sampling of the node positions were binned in 25 µm intervals and averaged (error bars indicating the degree of dispersion via the standard deviation). (C) In accordance with the strain rate profile (A) (which is the derivate of the velocity profile), the spatial profile of the longitudinal velocity at 50 h simulation time shows a sharp transition at around 200 µm from the QC (indicated by ‘*’). Data were derived from changes in cell positions over time (at 50 h and 55 h). (D) Velocity profile as in (C), but obtained through binning and averaging data from ten randomized simulations. The shape of the curve is smoother than in (C). The data points were fitted with a progress curve using the ‘polyloc’ method (cf. Methods). (TIF) [file pcbi.1003910.s005.tif]

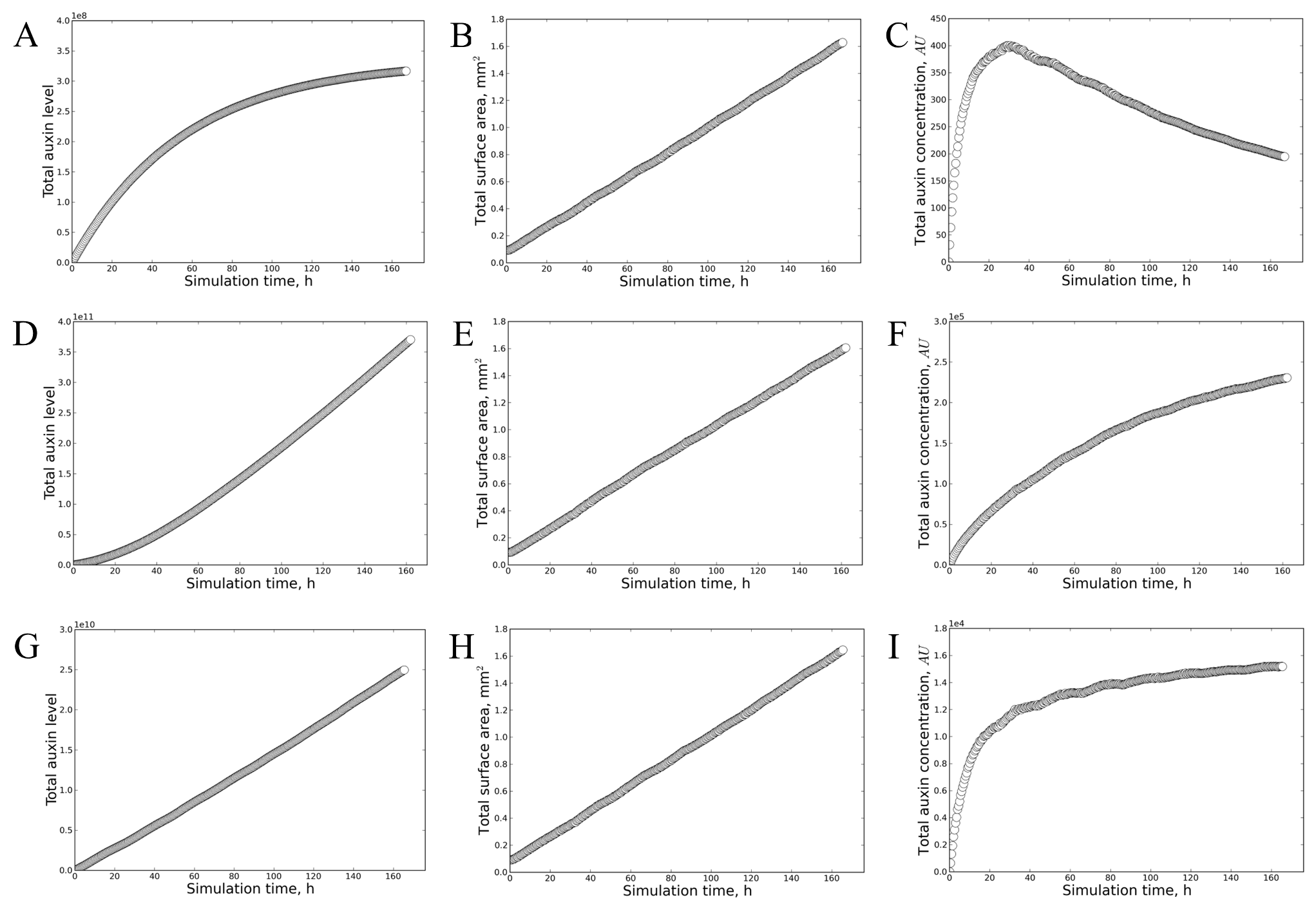

Supplement: Figure S5 — Temporal auxin patterns in a growing root. Evolution of the total auxin levels (A,D,G), the total simulated root area (B,E,H), and the auxin concentrations (C,F,I), corresponding to Figure 6A–C. A strictly external source of auxin combined with local sinks results in saturation of the total auxin in the root (A), which together with a steady increase in surface area (B) leads to the auxin concentration converging to zero (C). A strictly internal auxin source (production rates proportional to cell areas) results in the total auxin level (D) increasing proportionally to the area increase (E), and the auxin concentration slowly converging to a steady state (F). A strictly internal auxin source (production rates constant per cell) results in similar patterns as for (D–F) with a steady state in auxin concentration (I) as a function of the total auxin level (G) and area (H). (TIF) [file pcbi.1003910.s006.tif]

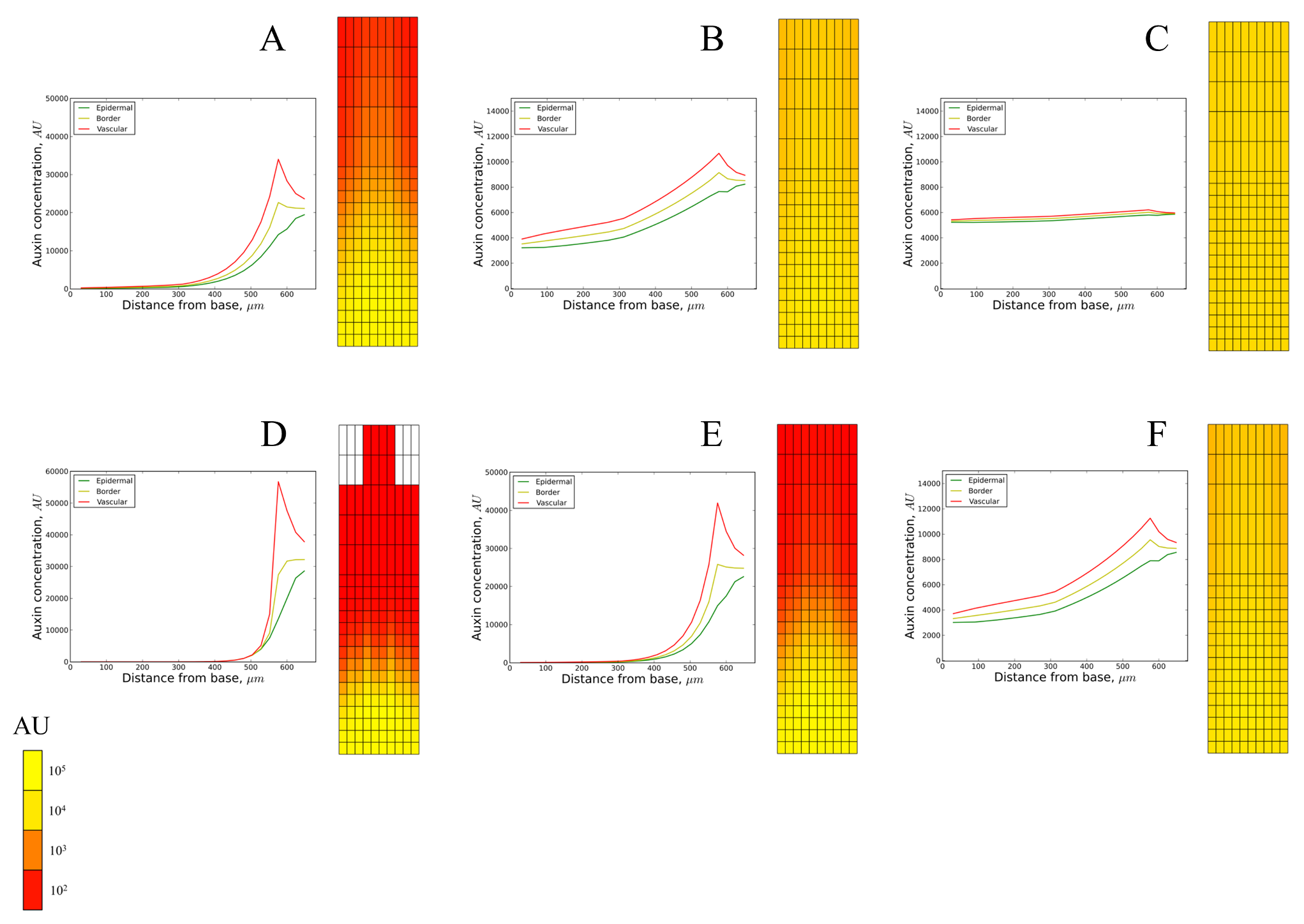

Supplement: Figure S6 — Effect of parameter variations on spatial auxin patterns I. Auxin concentration versus distance from the base of a simulated root for different cell files (2nd, 3rd, 5th from left) in a static mesh with variations in the diffusion constant D and the PIN exporter rate constant . The corresponding tissues with their auxin concentration pattern were included (yellow colouring; arbitrary units: AUs). (A) D = 1000 µm2/min, = 1200 µm/min; (B) D = 6000 µm2/min, = 1200 µm/min; (C) D = 36000 µm2/min, = 1200 µm/min; (D) D = 1000 µm2/min, = 7200 µm/min; (E) D = 6000 µm2/min, = 7200 µm/min; (F) D = 36000 µm2/min, = 7200 µm/min. Increasing D (compare A–C and D–F) flattens the auxin gradient, whereas increasing sharpens the gradient. Note that similar ratios of (such as in A and E, and B and F) lead to similar output. Fixed parameter values: = 600 µm/min, local auxin production = 10 AU/min, net auxin flux of top row = 106/min, apoplast thickness = 1 µm. As in Figure 6D a transversal gradient of auxin is visible. (TIF) [file pcbi.1003910.s007.tif]

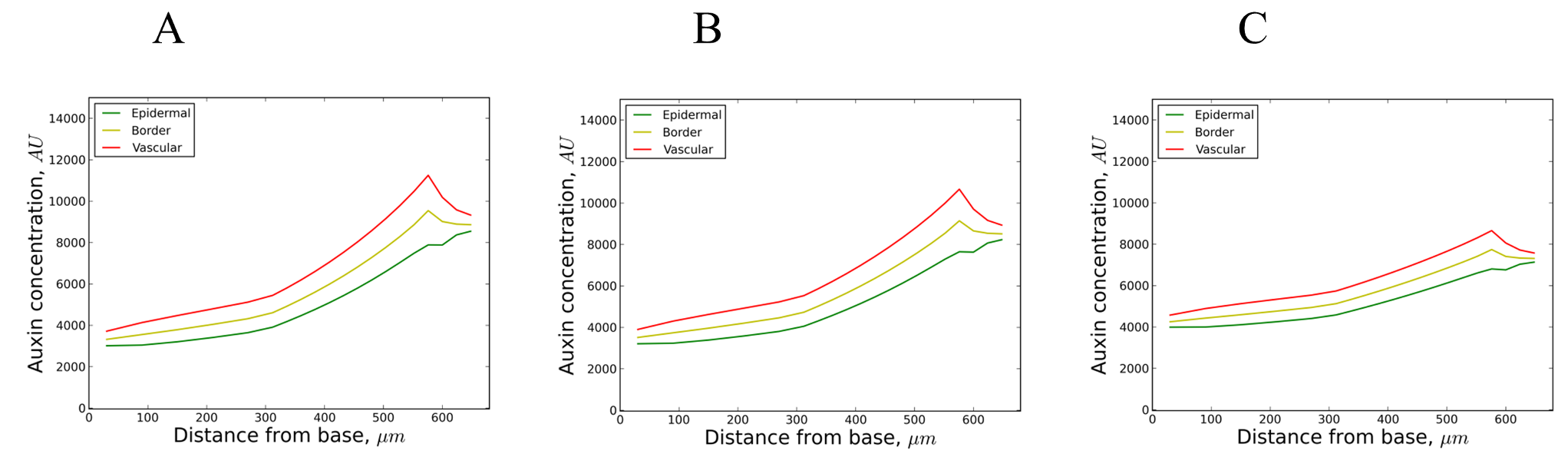

Supplement: Figure S7 — Effect of parameter variations on spatial auxin patterns II. Auxin concentration versus distance from the base of a simulated root for different cell files (2nd, 3rd, 5th from left) in a static mesh with variations in the importer rate constant . (A) = 100 µm/min; (B) = 600 µm/min; (C) = 3600 µm/min. The other parameters are the same as in Fig. S6B. Increasing to sufficiently high values, as with the diffusion coefficient D, flattens the auxin gradient. (TIF) [file pcbi.1003910.s008.tif]

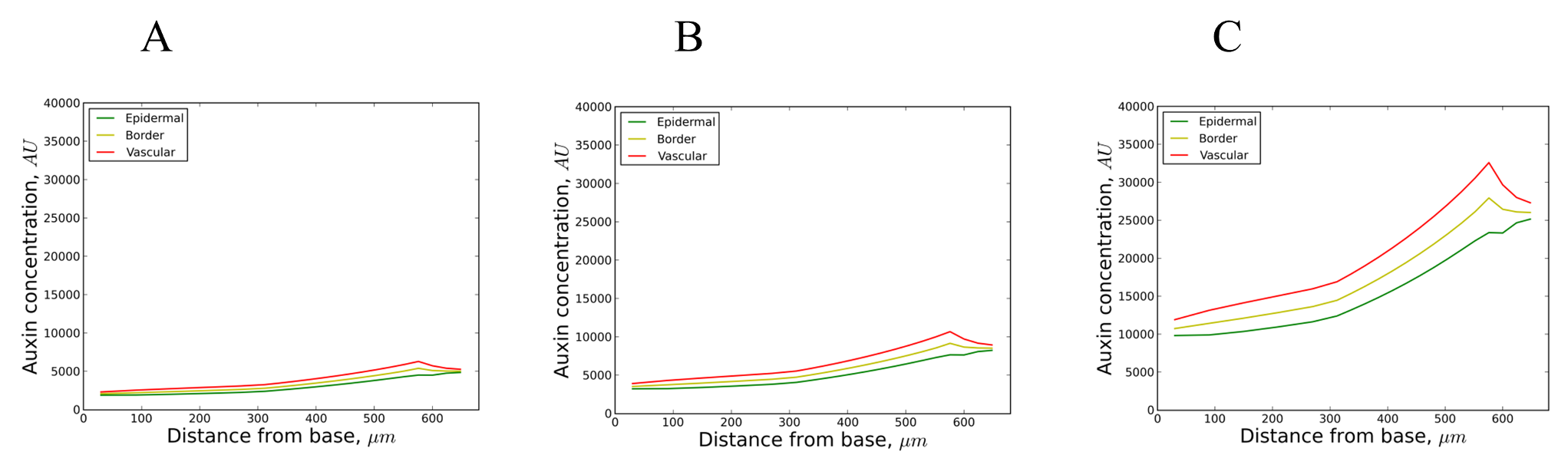

Supplement: Figure S8 — Effect of parameter variations on spatial auxin patterns III. Auxin concentration versus distance from the base of a simulated root for different cell files (2nd, 3rd, 5th from left) in a static mesh with variations in the local production rate . (A) = 2 µm/min; (B) = 10 µm/min; (C) = 50 µm/min. The other parameters are the same as in Fig. S6B. Increasing to sufficiently high values amplifies the overall auxin gradient. (TIF) [file pcbi.1003910.s009.tif]

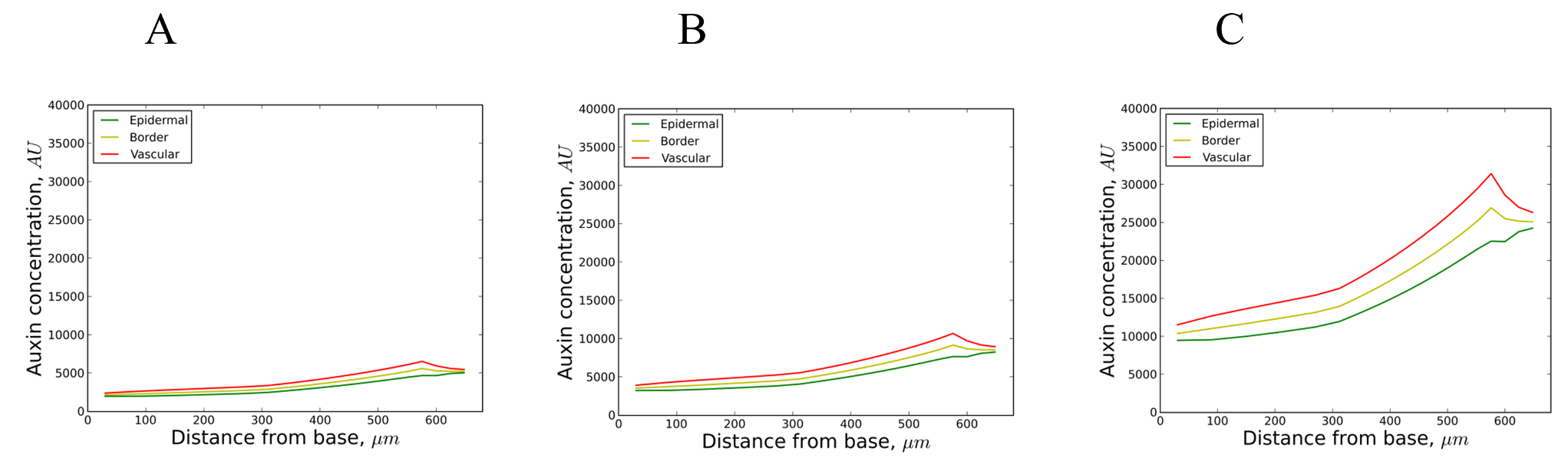

Supplement: Figure S9 — Effect of parameter variations on spatial auxin patterns IV. Auxin concentration versus distance from the base of a simulated root for different cell files (2nd, 3rd, 5th from left) in a static mesh with variations in the net total auxin influx of the top row of cells F′. (A) F′ = 2.105 1/min; (B) F′ = 106 1/min; (C) F′ = 5.106 1/min. The other parameters are the same as in Fig. S6B. Increasing F′ to sufficiently high values amplifies the overall auxin gradient. (TIF) [file pcbi.1003910.s010.tif]

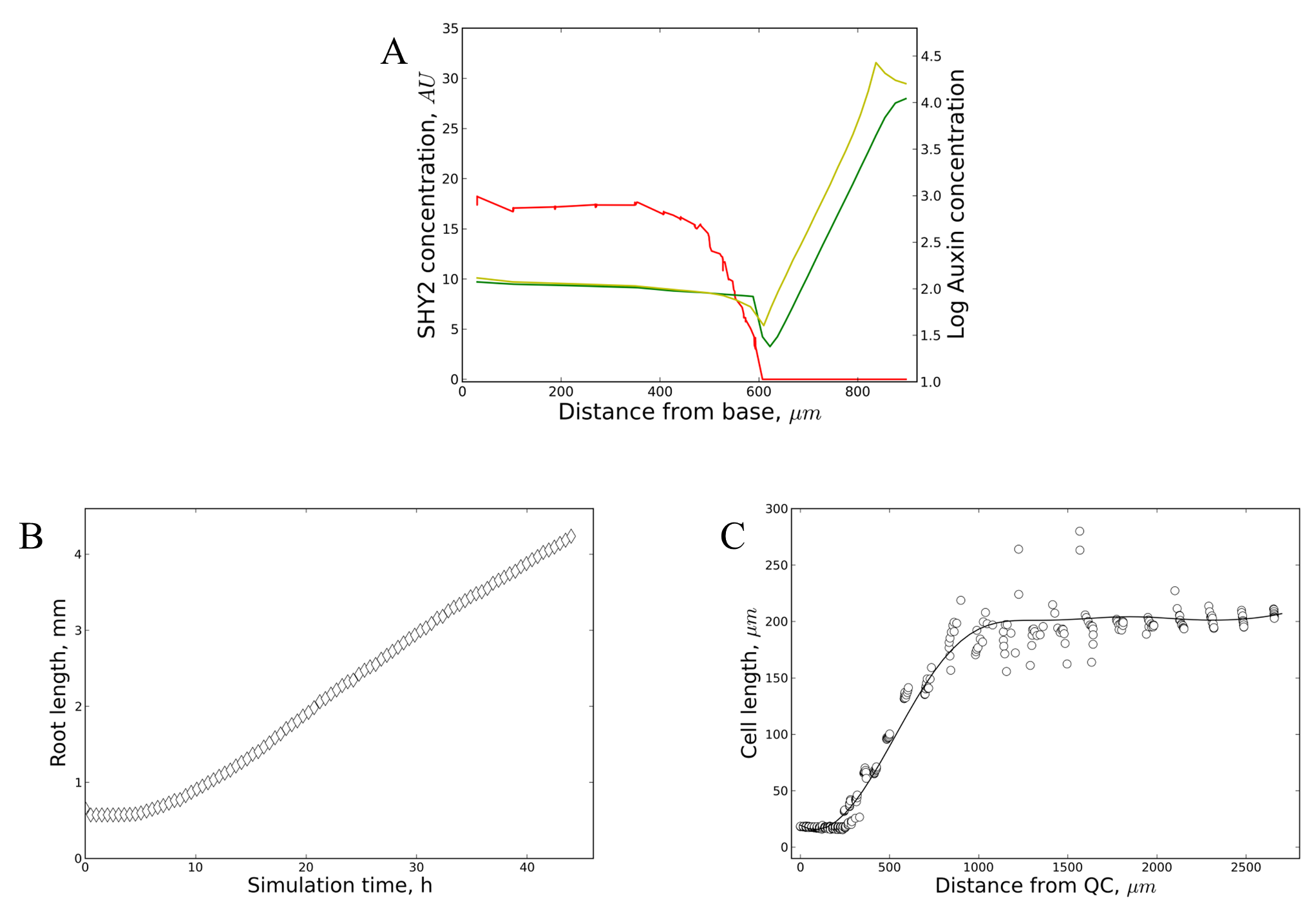

Supplement: Figure S10 — Model 12 leads to stable root growth with smooth transition in cell length obtained through SHY2 action. Simulation output demonstrates various aspects of Model 12. (A) Chemical concentration profiles (after 10 h simulation) across the 8th cell file (from the left) of SHY2 (red, AUs) and auxin (yellow, log10 values as dimensions). For auxin the profile is also plotted for the first cell file (from the left, coloured green, log10 values) to demonstrate the flattening effect of SHY2 on the auxin gradient. (B) Plot of root length versus simulation time showing steady linear organ growth after approximately 9 hours. (C) Plot depicting the cell length along the principal growth axis (at 60 h). Note that cell lengths vary smoothly from DZ to EZ (some synchronicity for cells at a similar axial position is visible, since no noise was added to the first divisions contrary to simulations pictured in for instance Fig. 5 ). (TIF) [file pcbi.1003910.s011.tif]

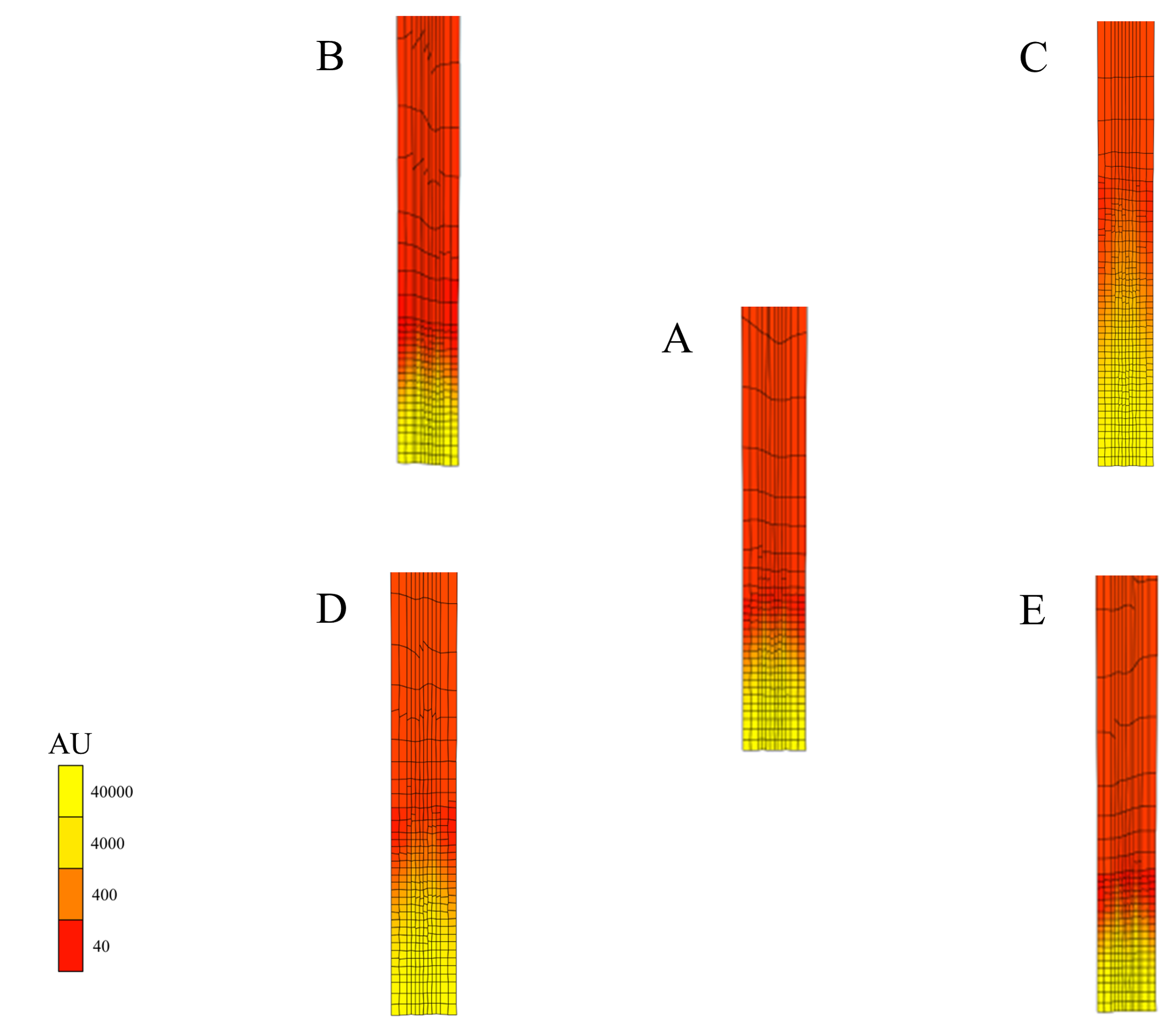

Supplement: Figure S11 — Effect of parameter variations on spatial auxin patterning in Model 12 . The simulation output of Model 12 (yellow colouring; arbitrary units: AU) is illustrated here for different parameter values of auxin diffusion (D) and a-polar transport (). (A) D = 900 µm2/min, = 2000 µm/min; (B) D = 600 µm2/min, = 2000 µm/min; (C) D = 3600 µm2/min, = 2000 µm/min; (D) D = 900 µm2/min, = 4000 µm/min. Increasing D (compare (B), (A), and (C)) expands the zone with high auxin activity and together with it the meristem, whereas increasing (compare (D), (A), and (E)) has the opposite effect. Note that keeping the constant (cf. Figure S6) should lead to similar output. Fixed parameter values were as in Table S2. (TIF) [file pcbi.1003910.s012.tif]

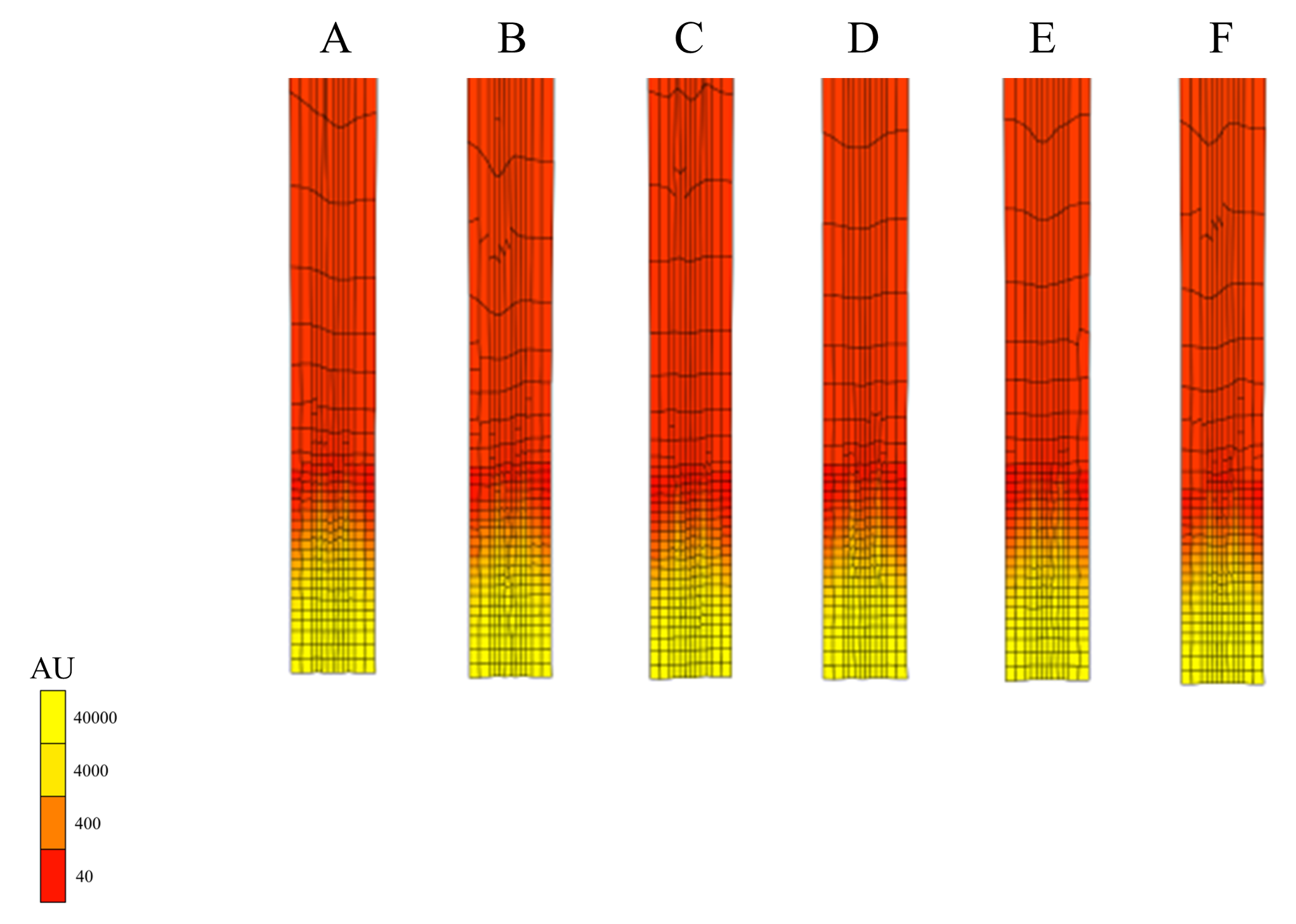

Supplement: Figure S12 — Model 12 is robust to small parameter variations. The simulation output of Model 12 (yellow colouring; arbitrary units: AU) is shown here for a 10% increase of different parameter values related to hormone transport: (A) simulation based on the reference parameter set (Table S2); (B) D[0] perturbed; (C) perturbed; (D) perturbed; (E) perturbed; (F) D[1] perturbed. The output is highly similar, which is also the case if these parameter values are decreased by 10% (results not shown), demonstrating local robustness/stability of the simulated output to changes of these parameters. (TIF) [file pcbi.1003910.s013.tif]
