# Supplementary material for: Putting Theory to the Test: Which Regulatory Mechanisms Can Drive Realistic Growth of a Root?
Source: PLoS Comput Biol. 2014 Oct 30;10(10):e1003910. doi: 10.1371/journal.pcbi.1003910 (PMC4214622; doi:10.1371/journal.pcbi.1003910)
Supplement: Table S1 — Model overview. Overview of the models used in this study. Various categories w.r.t. developmental decisions are presented. Column (3) specifies the transition between division and elongation zone (DZ and EZ, respectively) with in parentheses the number of division or time since release form the QC; column (4) specifies the transition to mature (differentiated) cells based on timing since the release from the QC or a spatial signal at a fixed distance from the root apex; column (5) specifies whether division rate is determined via a timer or sizer mechanism; and column (6) how cellular growth rates are defined. Developmental events can be determined to happen after a fixed duration (‘Timer’), a fixed number of divisions (‘Counter’), a fixed cell size (‘Sizer’), and a fixed distance from the root apex (‘Positional’, i.e. a ruler). For Models 10–12 more complicated regulatory mechanisms are specified. In Model 4 extra random noise was added to the timer (+/− max. 25%). For Model 5 cell division is dependent on a timer mechanism, with a different cell cycle time for inner and outer layers. Model 6 uses a uniform size (area) criterion for cell division, irrespectively of geometry. For Model 7 the size criterion is adapted to the difference in the width between inner and outer layers, thereby acting effectively as a length criterion. In the other (non-cell autonomous) cases the cell division sizers differs between layers. It is usually assumed that DZ and EZ have a characteristic elongation rate (expressed as a constant addition to the target area or a fraction of the current cell area). Layer-specific differences are indicated (by column numbers) if necessary. All models are identical in that the columella/QC cells (lower 3 cell rows) cannot divide. For timer and counter based models the contact with the QC cells is important to keep them dividing. As soon as they lose contact the respective counter or timer starts to increase. For non-cell-autonomous models a morphogen [file pcbi.1003910.s014.docx]

**Table S1. Model overview.**

Overview of the models used in this study. Various categories w.r.t. developmental decisions are presented. Column (3) specifies the transition between division and elongation zone (DZ and EZ, respectively) with in parentheses the number of division or time since release form the QC; column (4) specifies the transition to mature (differentiated) cells based on timing since the release from the QC or a spatial signal at a fixed distance from the root apex; column (5) specifies whether division rate is determined via a timer or sizer mechanism; and column (6) how cellular growth rates are defined. Developmental events can be determined to happen after a fixed duration (‘Timer’), a fixed number of divisions (‘Counter’), a fixed cell size (‘Sizer’), and a fixed distance from the root apex (‘Positional’, *i.e.* a ruler). For Models 10-12 more complicated regulatory mechanisms are specified. In Model 4 extra random noise was added to the timer (+/- max. 25%). For Model 5 cell division is dependent on a timer mechanism, with a different cell cycle time for inner and outer layers. Model 6 uses a uniform size (area) criterion for cell division, irrespectively of geometry. For Model 7 the size criterion is adapted to the difference in the width between inner and outer layers, thereby acting effectively as a length criterion. In the other (non-cell autonomous) cases the cell division sizers differs between layers. It is usually assumed that DZ and EZ have a characteristic elongation rate (expressed as a constant addition to the target area or a fraction of the current cell area). Layer-specific differences are indicated (by column numbers) if necessary. All models are identical in that the columella/QC cells (lower 3 cell rows) cannot divide. For timer and counter based models the contact with the QC cells is important to keep them dividing. As soon as they lose contact the respective counter or timer starts to increase. For non-cell-autonomous models a morphogen threshold determines if cells keep dividing. For each model cell division occurs through the insertion of a new horizontal wall dividing the cell in half. For models using hormone transport parameter values were used in accordance with known modelling studies such as [12, 17, Wabnik et al., 2010]. In general reaction and transport equations are discussed in Text S1. A detailed parameter description can be found in the respective figures or in Table S2.

Wabnik K, Kleine-Vehn J, Balla J, Sauer M, Naramoto S, Reinöhl V, Merks RM, Govaerts W, Friml J. (2010) Emergence of tissue polarization from synergy of intracellular and extracellular auxin signaling. Mol Syst Biol. 6:447.

| **Model** | **Fig.** | **Exit DZ -Start EZ** | **Exit EZ – Start MZ** | **Division rule** | **Growth rate (per simulation step of 30 min)** |
| --- | --- | --- | --- | --- | --- |
| 1 | 1C | Counter (2) | Timer (72h) | Timer (18h) | Constant (in µm^2^: DZ 2; EZ 20) |
| 2 | 3A-D | Timer (54h) | Timer (72h) | Id. | Linear (as a fraction: DZ 0.02; EZ 0.2) |
| 3 | S1A | Counter (3) | Timer (72h) | Id. | Id. |
| 4 | 4A | Timer (54h) | Timer (72h) | Timer (18h) +/- max. 25% | Linear (fract.: DZ 0.02; EZ 0.1) |
| 5 | 4B | Counter (3) | Timer (74h) | Timer ( outer 6: 15h; inner 6: 18h) | Linear (fract.: DZ 0.02; EZ 0.2) |
| 6 | 4C | Counter (2) | Timer (72h) | Sizer (outer 6 = inner 6 = 80 µm^2^) | Linear (fract.: DZ 0.02; EZ 0.2) |
| 7 | S3B | Counter (3) | Timer (72h) | Sizer (outer 4: 160 µm^2^; inner 8: 80 µm^2^) | Linear (fract.: DZ 0.02; EZ 0.1) |
| 8 | 5A-D | Positional signal I (180 µm) | Positional signal II (800 µm) | Sizer (outer 4 = 240 µm^2^; border=144 µm^2^: inner 6 = 120 µm^2^) | Linear (fract.: DZ 0.02; EZ 0.4) |
| 9 | 6A-D | Positional signal I^*^ | Positional signal II (750 µm) | Sizer (400 µm^2^) | Linear (fract.: DZ 0.02; EZ 0.2) |
| 10 | 7A,B | Auxin threshold (7A: 13.5 AU,  7B: 60,000 AU) | Auxin  Threshold  (7A: 8.8 AU,  7B: 40,000) | Sizer (outer 4= 160 µm^2^; border= 80 µm^2^; inner= 96 µm^2^) | Linear (fract.: DZ 0.02; EZ 0.2) |
| 11 | 8 | Layer-specific auxin threshold  (8 AU) | Layer-specific auxin threshold  (6 AU) | Sizer (outer 4= 160 µm^2^; border= 80 µm^2^; inner 6= 96 µm^2^) | Linear (fract.: DZ 0.02; EZ 0.2) |
| 12 | 9 | SHY2 threshold  (>0.1 AU) | GA threshold  (<0.7 AU) | Sizer (outer 4: 400 µm^2^, inner 8: 200 µm^2^) | Linear (fract.: DZ 0.02; EZ 0.2) |

*Two different signal for proliferation exit (180 µm) and start acceleration (240 µm).
