# Supplementary material for: Putting Theory to the Test: Which Regulatory Mechanisms Can Drive Realistic Growth of a Root?
Source: PLoS Comput Biol. 2014 Oct 30;10(10):e1003910. doi: 10.1371/journal.pcbi.1003910 (PMC4214622; doi:10.1371/journal.pcbi.1003910)
Supplement: Table S2 — Overview of kinetic parameters of Model 12 . For Model 12 the auxin reaction and transport parameters D[0], k_export, and k_import, were adjusted to produce an auxin gradient with a more pronounced and central maximum such as found in various reporter studies (see for instance Fig. 3c in [12] and Fig. 1A in [18]). Figures S11 and S12 illustrate parameter dependent behaviour and sensitivity. The model behaviour is robust to hormone transport parameters changes and even larger changes can in principle be accommodated based on the balance between a-polar and polar auxin transport and stable negative feedback regulation of auxin and cytokinin. (DOCX) [file pcbi.1003910.s015.docx]

**Table S2. Overview of kinetic parameters of *Model 12.***

| **Parameter** | **Value [Units]** | **Explanation** |
| --- | --- | --- |
| D[0] | 900 [µm^2^/min] | Auxin diffusion constant |
| D[1] | 12 [µm^2^/min] | Cytokinin diffusion constant |
| apoplast_thickness | 1 [µm] | Apoplast thickness |
| k_import | 60 [µm/min] | Auxin import permeability |
| k_export | 2000 [µm/min] | Auxin export permeability |
| km_shy | 0.1 [AU*] | Shy2-dependent auxin export inhibition constant |
| aux_production | 1 [AU/min] | Cellular auxin production rate constant |
| aux_breakdown | 1.5e-3 [1/min] | Cellular auxin breakdown rate constant |
| aux_source | 1e5 [1/min] | Rate constant for incoming auxin flux |
| aux_sink | 1e5 [1/min] | Rate constant for outgoing auxin flux |
| ck_source | 25 [1/min] | Rate constant for incoming cytokinin flux |
| ck_sink | 25 [1/min] | Rate constant for outgoing cytokinin flux |
| vm_aux_ck | 0.01 [AU/min] | Auxin-dependent cytokinin production rate constant |
| km_aux_ck | 100 [AU] | Auxin-dependent cytokinin inhibition constant |
| ck_breakdown | 5e-4 [1/min] | Cellular cytokinin breakdown rate constant |
| shy2_production | 1 [AU/min] | Cellular shy2 production rate constant |
| shy2_breakdown | 1e-3 [1/min] | Cellular shy2 breakdown rate constant |
| aux_shy2_breakdown | 0.1 [1/min] | Auxin-dependent shy2 breakdown rate constant |
| km_aux_shy2 | 100 [AU] | S_0.5_ for auxin-dependent shy2 breakdown |
| ga_production | 100 [1/min] | Cellular gibberellin production rate constant |
| ga_breakdown | 0.1 [1/min] | Cellular gibberellin breakdown rate constant |

* ‘AU’: Arbitrary concentration Units
