# Supplementary material for: Putting Theory to the Test: Which Regulatory Mechanisms Can Drive Realistic Growth of a Root?
Source: PLoS Comput Biol. 2014 Oct 30;10(10):e1003910. doi: 10.1371/journal.pcbi.1003910 (PMC4214622; doi:10.1371/journal.pcbi.1003910)
Supplement: Text S1 — Background information on model implementation. (DOCX) [file pcbi.1003910.s016.docx]

**Text S1. Model implementation.**

All model-specific C++ code is added as supporting material (Dataset 1) and simulations can be reproduced using the open source (GNU General Public License) software VirtualLeaf which is available at <http://virtualleaf.googlecode.com> (more information can be found in [25]). Besides methods describing the rules for cell division, cell properties inheritance by daughter cells, cell growth, cell-to-cell transport and cell chemistry, an xml-based input file is necessary to specify the mechanical, numerrical integration and biochemical parameter values as well as the starting state of the cellular grid or mesh. Briefly (see Methods for more information), cells are described by nodes with have attributes such as their position. The nodes interconnect to form walls and cells which also have various attributes such as the levels of its chemicals. Three example xml files representative of the three types of starting geometry found across all models can be found in Dataset 1. Dataset 1 also contains a file called ‘Wortel.cpp’ which represents the so called model plugin containing detailed code describing division, growth and chemical behaviour for all twelve models. Since the file is extensive we have picked a few cases out to explain here in a bit more detail.

The first examples demonstrate how the *CellHouseKeeping* method, which serves to describe cell division and cell growth, differs between the various model types. After each Monte Carlo equilibration cycle each cell will evaluate the rules within this method.

*TIMER-based model implementation (for both the cell cycle and the exit from proliferation):*

Each cell will call the simulation time and the time since the last division of that cell and then evaluate how much time has passed. If this number is above the pre-set cell cycle time (1080 minutes in the example) multiplied by a randomizing factor that represents noise in cellular behaviour, then division is allowed under certain conditions. The first condition is if cells are in contact with the ‘columella/QC’ cells (lower 3 cell rows of mesh). In this case a second timer is reset to zero upon division to represent its stem cell character (indefinite division potential). In the second conditional expression the time since the last reset of the latter timer is tested and division is allowed if not more than 3240 minutes have passed since the cell was disconnected from the QC through division. This represents cell behaviour in the division zone.

// CURRENT TIME MEASURED

time_now = m_mesh->GetSimTime();

// RANDOM NUMBER GENERATED 1 +/- 10%

double cc_noise = 1 + (RANDOM()-0.5) / 5;

// EVALUATE TIME SINCE DIVISION

if ( ( time_now - c->LastDivisionTime()) >= ( 1080 * cc_noise ) )

{

// EVALUATE CONTACT WITH ‘QC’

if ( c->HasNeighborOfTypeZero() )

{

// RESET TIME SINCE ENTERING PROLIFERATIVE PHASE

c->SetProliferationTime( m_mesh->GetSimTime() );

// DIVIDE OVER HORIZONTAL AXIS

c->DivideOverAxis(Vector(1,0,0));

}

// EVALUATE TIME SINCE PROLIFERATION

else if ( ( time_now - ( c->GetProliferationTime() ) ) <= 3240

{

// DIVIDE OVER HORIZONTAL AXIS

c->DivideOverAxis(Vector(1,0,0));

}

}

In the next example, instead of a timer the cell will call a function to know if it is in contact with the ‘QC’ cells (of designated type zero) or if it has divided less than four times since it was disconnected from the QC. In that case slow growth is specified through an update of the target area of the cell by 0.02 times its current area. In the other cases a second condition evaluates how much time has passed since the last division (timer). If below 5280 then fast growth (factor 0.2) is specified in accordance with elongation zone behaviour.

*Implementation of simple COUNTER mechanism to regulate expansive growth:*

// EVALUATE CONDITION FOR PROLIFERATIVE STAGE

if ( (c->HasNeighborOfTypeZero() OR ( c->NumberOfDivisions2() <= 3 ))

{

// LINEAR GROWTH RATE: SLOW

c->EnlargeTargetArea( 0.02 * ( c->Area() ) );

}

// EVALUATE CONDITION FOR EXPANSIVE GROWTH

else if (( c->NumberOfDivisions2() > 3 ) AND ( time_now - c->GetDivisionTime() ) <= 5280.)

{

// LINEAR GROWTH RATE: FAST

c->EnlargeTargetArea( 0.2 * ( c->Area() ) );

}

*Implementation of a prototypical SIZER:*

Each file of cells has been designated a type (specified in the input xml file) based on its horizontal position and its PIN distribution (this is important to allow polarity to be propagated to the daughter cells upon division). In the example, the inner six layers (types 4-6) will divide if their size is above 80 µm^2^ (multiplied with a factor for noise) and if they are in contact with the QC or if they have divided less than four times. If cells remain in contact with the QC, then the division counter is reset to zero as well as a timer that indicates the time since detachment from the QC. The outer cell layers (of types 1,2,8,9) and ‘border’ cell layers (types 3 and 7) have a different size upon division (160 µm^2^ and 96 µm^2^, respectively). The rest of the conditions are the same (not shown).

// EVALUATE CELL SIZE OF INNER CELL LAYERS (UNIFORM WHITE NOISE ADDED)

if ( ( c->CellType() > 3 AND c->CellType() < 7 ) AND ( c->Area() >= ( 80. * cc_noise) ))

{

//CELLS IN CONTACT WITH QC CAN DIVIDE

if ( c->HasNeighborOfTypeZero() )

{

// RESET DIVISION COUNTER

c->SetDivCounter2( 0 );

//RESET TIME SINCE PROLIFERATION

c->SetProliferationTime( m_mesh->GetSimTime() );

c->DivideOverAxis(Vector(1,0,0));

}

//CELLS WHICH HAVE DIVIDED A LIMITED NUMBER OF TIMES CAN DIVIDE

else if ( c->NumberOfDivisions2() <= 3 )

{

c->DivideOverAxis(Vector(1,0,0));

}

}

// EVALUATION FOR OUTER CELL LAYERS

if ( c->CellType() != 0 AND ( c->CellType() > 7 OR c->CellType() < 3 ) AND

( c->Area() >= ( 160.* cc_noise) ) )

{

…

}

// CONDITION FOR CELL TYPES 3 AND 7 (‘BORDER’)

if ( ( c->CellType() == 3 OR c->CellType() == 7 ) AND ( c->Area() >= ( 96.* cc_noise ) ) )

{

…

}

*Non-cell-autonomous mechanisms* include the following equations for auxin dynamics (combined here but implemented in the separate *CelltoCellTransport* and *CellDynamics* methods of the model plugin):

(Eq.4)

With:

$c_{i}$: Auxin concentration in cell i;

$j$: Neighbouring cell index;

$A_{i}$: Surface area of cell i;

$L_{ij}$: Wall segment length between cell i and j;

$T$: Wall thickness;

$D$: Diffusion constant;

$k_{ex}$: PIN exporter rate constant;

$\delta_{ji}$: = 1/0 depending on presence/absence of PIN exporter from cell j to cell i;

$k_{im}$: AUX1 importer rate constant;

$k_{d}$: Auxin degradation rate constant;

$\sigma$: Cellular auxin production rate;

$F$: Auxin influx/efflux rate (only in upper cell row).

Transport equations:

Diffusion is expressed by the first summation term of equation (4) based on Fick’s diffusion law. This expression (with varying diffusion coefficient D) was used for any diffusible substance (see for instance the code example of *Model 12*).

PIN exporter and AUX/LAX importer-kinetics is expressed by the second summation term of equation (4). It describes export from cell *i* as the sum of individual export processes to all neighbouring cells. It is proportional to the cell’s auxin concentration and $k_{ex}$(permeability constant) and is also multiplied by a polarity factor $\delta_{ji}$which describes the presence (1) or absence (0) of PIN in the walls separating the respective neighbouring cells. Furthermore, the export from all neighbouring cells to cell *i* is also included. Since importer distribution is not polar, an extra $k_{im}$-dependent term is added to each export process (independent from the value of the $\delta_{ji}$’s). In the actual implementation (illustrated in the next section) cell-to-cell transport is effected by cycling over all ‘walls’ (a ‘wall’ representing the boundary between two cells) and evaluating all transport processes accordingly such that each process is accounted for only once.

Auxin degradation was in all cases expressed by first order decay. Other sources and sinks were as follows:

Local auxin production was either proportional to the cell area (area-based) or constant for each cell: in the first case a constant auxin production term $\sigma$is added to the differential equation. In the second (cell-based) case a constant auxin production term $\sigma$divided by the cell area is added to the differential equation.

External supply and demand were specified through additional terms for the top (non-growing) cell row. If cell *i* belongs to the first or last 2 cells of the highest cell row, then it acts as a sink via a constant flux *F_out_*. If it belongs to the other cells of that row then it acts as a constant source *F_in_.*

The parameter dependent behaviour of equation (4) was extensively tested (Figures S6-S9 for details) and robustly represents hormone dynamics on a two-dimensional cellular grid. The time-dependent behaviour of a typical auxin gradient was studied as well (Figure 6).

Similar diffusion and production/supply kinetics have been used for cytokinin in *Model 12*. Detailed equations for other central processes in that model can be found below. Table S2 contains additional parameter values and explanations. Figures S11 and S12 demonstrate robust behaviour of *Model 12* under variation of parameters for auxin and cytokinin dynamics.

*Auxin and cytokinin transport implementation:*

In accordance with the previous section cell-to-cell transport is evaluated for all walls in each simulation step (time step) of the model. In contrast to auxin, cytokinin transport is assumed to happen only through diffusion. The following code fragments illustrate how the respective differential equations are described and parameterized in the implementation of the plugin. Importantly, the wall-specific processes affect the cell based differential equation (for time dependent changes of numbers/levels of chemicals, not concentration) for instance for auxin through the variables dchem_c1[0] and dchem_c2[0]. Noteworthy is that PIN presence in walls is evaluated via the Transporters1() function. In this model PIN mediated transport is inhibited via the cellular SHY2 concentration by multiplying the export rate expression with

$\frac{km\_shy}{km\_sky+[SHY2]}$ (function value varying from 0 to 1, depending on [SHY2]).

//DIFFUSIVE AUXIN (CHEMICAL 0) TRANSPORT FLUXES FOR CELL 1 AND 2 BORDERING //WALL W

double const apoplast_thickness = par.apoplast_thickness;

double const phi = (w->Length() / apoplast_thickness) * par.D[0] *

( ( w->C2()->Chemical(0) / (w->C2()->Area()) ) - ( w->C1()->Chemical(0) / (w->C1()->Area()) ) );

// AUXIN NUMBER TRANSPORT ODES FOR CELL 1 AND CELL 2: DIFFUSION

dchem_c1[0] += phi;

dchem_c2[0] -= phi;

//MEDIATED AUXIN TRANSPORT

double const k_import = par.k_import;

double const k_export = par.k_export;

double const km_shy = 0.1;

//INHIBITORY EFFECT ON PIN MEDIATED EXPORT FROM CELL 1

//SHY2 = CHEMICAL 2, SHY12 IN [0,1]

double const shy12 = km_shy / ( km_shy + ( w->C1()->Chemical(2) ) / (w->C1()->Area()) );

//MEDIATED TRANSPORT RATE FROM CELL 1 TO CELL 2

double const trans12 = w->Length() * ( w->C1()->Chemical(0) / (w->C1()->Area()) ) *

(k_export * w->Transporters1(1) * shy12 + k_import);

//INHIBITORY EFFECT ON PIN MEDIATED EXPORT FROM CELL 2

//SHY21 IN [0,1]

double const shy21 = km_shy / ( km_shy + ( w->C2()->Chemical(2) ) / (w->C2()->Area()) );

//MEDIATED TRANSPORT RATE FROM CELL 2 TO CELL 1

double const trans21 = w->Length() * ( w->C2()->Chemical(0) / (w->C2()->Area()) ) * (k_export * w->Transporters2(1) * shy21 + k_import);

//AUXIN TRANSPORT ODES FOR CELL 1 AND CELL 2: MEDIATED TRANSPORT

dchem_c1[0] += (trans21 - trans12);

dchem_c2[0] += (trans12 - trans21);

//DIFFUSIVE CYTOKININ (CHEMCIAL 1) TRANSPORT FLUXES FOR CELL 1 AND 2 //BORDERING WALL W

double phi2 = (w->Length() / apoplast_thickness) * par.D[1] *

( ( w->C2()->Chemical(1) / (w->C2()->Area()) ) - ( w->C1()->Chemical(1) / (w->C1()->Area()) ) );

//CYTOKININ NUMBER TRANSPORT ODES FOR CELL 1 AND CELL 2: DIFFUSION

dchem_c1[1] += phi2 ;

dchem_c2[1] -= phi2 ;

*Intracellular dynamics of auxin, cytokinin, SHY2 and gibberellin in Model 12:*

The following lines of code describe how the differential equations for chemical levels (not concentrations) in each cell (represented by the variables dchem[*i*]) are defined. Depending on the cell index the production rate of auxin and cytokinin can differ, with extra sources and sinks present for cells of the top row of the mesh (cell indices 0 to 11). Apart from those exceptions, the rest of the cells have production (area-based) and degradation (first order kinetics) terms. Importantly, cytokinin production is limited by the cellular auxin concentration by multiplying the production rate $vm\_aux\_ck$ with:

$$\frac{km\_aux\_ck}{km\_aux\_ck+[AUX]}$$

SHY2 production is cytokinin-dependent: only occurring if the latter concentration reaches 2.5 AU. On the other hand SHY2 is degraded in an auxin-dependent manner by describing its breakdown rate as:

$shy2\_breakdown+aux\_shy2\_breakdown\cdot\frac{[AUX]}{km\_aux\_shy2+[AUX]}$.

GA production (on a per cell basis) was assumed to be 2-fold higher for the wider cell rows to allow working with one GA concentration threshold (see next sections) as the criterion to stop cell elongation.

//CELLULAR DYNAMCIS OF CHEMICAL 0 (AUXIN) AND CHEMICAL 1 (CYTOKININ)

//8 INNER CELLS OF TOP CELL ROW REPRESENT AUXIN AND CYTOKININ SOURCES

//AS IF COMING FROM UPPER PARTS OF PLANT

if (c->Index() >= 2 AND c->Index() <= 9 )

{

//ODES FOR AUXIN AND CYTOKININ LEVELS

dchem[0] = par.aux_source + (c->Area()) * par.aux_production

- par.aux_breakdown * (c->Chemical(0)) ;

dchem[1] = par.ck_source + ( par.vm_aux_ck * (c->Area()) * par.km_aux_ck / ( par.km_aux_ck +

(c->Chemical(0) / (c->Area())) ) ) – par.ck_breakdown * (c->Chemical(1));

}

//4 OUTER CELLS ARE AUXIN AND CYTOKININ SINKS

else if ( (c->Index() = 0 OR c->Index() = 1 OR c->Index() = 10 OR c->Index() = 11 )

{

dchem[0] = -par.aux_sink + (c->Area()) * par.aux_production

- par.aux_breakdown * (c->Chemical(0));

dchem[1] = -par.ck_sink + (par.vm_aux_ck * (c->Area()) * par.km_aux_ck / ( par.km_aux_ck +

(c->Chemical(0) / (c->Area())) ) ) – par.ck_breakdown * (c->Chemical(1)) ;

}

//REST OF THE CELLS…

else

{

dchem[0] = (c->Area()) * par.aux_production - par.aux_breakdown * (c->Chemical(0)) ;

dchem[1] = (par.vm_aux_ck * (c->Area()) * par.km_aux_ck / (par.km_aux_ck + (c->Chemical(0)

/ (c->Area())) ) ) – par.ck_breakdown * (c->Chemical(1)) ;

}

//CHEMICAL 2 (SHY2) DYNAMICS

//SHY2 PRODUCTION ONLY ABOVE A CYTOKININ CONCENTRATION THRESHOLD

if( ( c->Chemical(1) / (c->Area()) ) > 2.5 )

{

// …INCLUDING AUXIN CONCENTRATION DEPENDENT SHY2 BREAKDOWN

dchem[2] = par.shy2_production * (c->Area()) - (c->Chemical(2)) * ( par.shy2_breakdown +

par.aux_shy2_breakdown * ( (c->Chemical(0) / c->Area()) / (par.km_aux_shy2 + c->Chemical(0) /

c->Area()) ) );

}

else

{

dchem[2] = - par.shy2_breakdown * (c->Chemical(2));

}

//CHEMICAL 3 (GA) DYNAMICS

//INNER, NARROW CELLS

if ( c->CellType() > 2 AND c->CellType() < 8 )

{

dchem[3] = par.ga_production – par.ga_breakdown * c->Chemical(3);

}

else

{

//OUTER, WIDER CELLS HAVE PROPORTIONALLY HIGHER PRODUCTION

dchem[3] = 2 * par.ga_production – par.ga_breakdown * c->Chemical(3);

}

*Spatially regulated cell division in Model 12:*

Cell division can occur only if a specific cell area has been reached. For the narrow inner cell layers (types 3-7) this is at 200 µm^2^ and for the wide outer cell layers 400 µm^2^. This mechanism conserves the cell length in the meristem. The principal necessary condition for cell division is the arbitrary SHY2 concentration threshold (0.1 AU). However, an extra criterion was included to represent that very low GA concentration values (below 0.7 AU) are exclusive for cell division (inspired by experimental work from [78]).

// GENERATE A RANDOM REAL FROM [0.9,1.1]

double cc_noise = 1 + (RANDOM()-0.5)/5;

//FIRST FIVE (FIXED) CELL ROWS DO NOT DIVIDE TO ALLOW SPONTANEOUS FORMATION OF A REALISTIC ROOT TISSUE

if ( c->Index() > 60 )

{

//DIVISION CRITERIA BASED ON CONCENTRATIONS OF CHEMICAL 2 (SHY2) AND 3 (GA)

if ( (( c->Chemical(2) / c->Area() ) < 0.1) AND (( c->Chemical(3) / c->Area() ) >= 0.7))

{

//’NOISY’ SIZE CRITERIUM FOR INNER (NARROWER) CELLS

if ( ( c->CellType() > 2 AND c->CellType() < 8 ) AND

( c->Area() >= ( 200. * cc_noise) ) )

{

c->DivideOverAxis(Vector(1,0,0));

}

//’NOISY’ SIZE CRITERIUM FOR OUTER (WIDER) CELLS

if ( c->CellType() != 0 AND ( c->CellType() > 7 OR c->CellType() < 3 ) AND

( c->Area() >= ( 400.* cc_noise) ) )

{

c->DivideOverAxis(Vector(1,0,0));

}

}

}

*Spatially regulated cell expansion in Model 12:*

The same SHY2 concentration determines whether growth will be slow (0.02 fractional increase in target area for SHY2 concentrations below 0.1) or fast (0.2 fractional increase in target area for values above 0.1).

//CHEMICAL 2 (SHY2) AND CHEMICAL 3 (GA) CONCENTRATION DEPENDENT EXPANSION

//FOR ALL EXCEPT CAP CELLS

if ( c->CellType() != 0 AND ( c->Chemical(3) / c->Area() ) >= 0.7)

{

//IF SHY2 BELOW THRESHOLD: SLOW GROWTH

if ( ( c->Chemical(2) / c->Area() ) < 0.1)

{

c->EnlargeTargetArea( 0.02 * ( c->Area() ) );

}

//OTHERWISE: FAST GROWTH

else

{

c->EnlargeTargetArea( 0.2 * ( c->Area() ) );

}

}
